# Evaluation of STK17B as a cancer immunotherapy target utilizing highly potent and selective small molecule inhibitors

**DOI:** 10.3389/fimmu.2024.1411395

**Published:** 2024-10-21

**Authors:** Felix Scheuplein, Florian Renner, John E. Campbell, Robert Campbell, Chris De Savi, Jan Eckmann, Holger Fischer, Jie Ge, Luke Green, Peter Jakob, Joseph L. Kim, Caitlin Kinkema, Katie McGinn, Ricardo Medina, Annemarie Müller, Nisha Perez, Emanuele Perola, Yoav Timsit, Tary Traore, Ulrike Hopfer, Stefka Tyanova, Manuel Tzouros, Ruduan Wang, Richard Woessner, Marion Dorsch, James R. Bischoff

**Affiliations:** ^1^ Blueprint Medicines Corporation, Cambridge, MA, United States; ^2^ Roche Innovation Center Basel, Basel, Switzerland; ^3^ Roche Innovation Center Munich, Penzberg, Germany

**Keywords:** DRAK2, STK17B, immunotherapy, small molecule inhibitors, T cells, kinase

## Abstract

**Introduction:**

The serine/threonine kinase 17B (STK17B) is involved in setting the threshold for T cell activation and its absence sensitizes T cells to suboptimal stimuli. Consequently, STK17B represents an attractive potential target for cancer immunotherapy.

**Methods:**

To assess the potential of STK17B as an immuno-oncology target, we developed potent and selective tool compounds from starting points in Blueprint Medicines Corporation's proprietary kinase inhibitor library. To characterize these molecules, enzyme and cellular assays for STK17A and STK17B were established to drive chemistry optimization. Mass spectrometry-based phosphoproteomics profiling with tool inhibitors led to the identification of Ser19 on myosin light chain 2 as STK17B substrate, which is then developed into a flow cytometry-based pharmacodynamic readout of STK17B inhibition both *in vitro* and *in vivo*.

**Results:**

In a mouse T cell activation assay, STK17B inhibitors demonstrated the ability to enhance interleukin-2 (IL-2) production. Similarly, treatment with STK17B inhibitors resulted in stronger cytokine secretion in human T cells activated using a T cell bispecific antibody. Subsequent chemistry optimization led to the identification of a highly selective and orally bioavailable tool compound, BLU7482. *In vivo*, STK17B inhibition led to dose-dependent modulation of myosin light chain 2 phosphorylation and enhanced priming of naïve T cells, as determined by upregulation of CD69, IL-2 and interferon-γ secretion. In line with increased T cell activation, treatment with STK17B inhibitor enhanced antitumor activity of anti–PD-L1 antibody in the MCA205 model.

**Conclusions:**

In summary, we successfully identified and optimized STK17B kinase inhibitors which led to increased T cell responses *in vitro* and *in vivo*. This allowed us to evaluate the potential of STK17B inhibition as an approach for cancer immunotherapy.

## Introduction

In the growing field of cancer immunotherapy, there has been interest in the roles of the death-associated protein kinase (DAPK) family of serine/threonine protein kinases (1, 2). The DAPK family is thought to be involved in several cell death-related signaling pathways, including regulation of apoptosis, autophagy, tumor suppression, and inhibition of metastasis, among others (1, 2). Serine/threonine kinase 17B (STK17B), also known as DAP kinase-related apoptosis-inducing protein kinase 2 (DRAK2), is a member of the DAPK family which has emerged as a potential cancer immunotherapy target. STK17B shows the highest expression in developing and mature lymphocytes (3–5), and previous studies have demonstrated its involvement in setting the threshold for T cell activation (3, 5–7). In human but not in mouse, STK17B has a closely related homologue STK17A (also called DRAK1) which is also a member of the DAPK kinase family. Interestingly, there was an evolutionary chromosomal re-arrangement event in rodents, such that they have lost the paralog STK17A gene completely and only have STK17B gene (8). STK17B and STK17A share 60% amino acid identity, however, the function of STK17A is not well understood. McGargill et al. have reported that deletion of the *STK17B* gene in mice renders T cells sensitive to suboptimal stimuli (3, 7). T cells from STK17B-deficient mice have been shown to have a lowered threshold for T cell receptor (TCR) activation, leading to increased priming by suboptimal antigens *in vitro* (3). While STK17B deficiency led to defective T cell survival in an experimental autoimmune encephalomyelitis model, STK17B-deficient mice did not show significant defects in the immune response to acute viral infection (6). STK17B was also identified in an *in vivo* CRISPR screen in tumor-bearing animals to identify novel cancer immunotherapy targets, where STK17B-depleted T cells were highly enriched in the tumor-infiltrating lymphocyte population (9).

These factors make STK17B an appealing putative immunotherapy target with the potential to enhance the efficacy of current immune checkpoint inhibitors. Targeting the programmed cell death protein 1/programmed death-ligand 1 (PD-1/PD-L1) axis in combination with lowering the threshold for suboptimal antigens by inhibiting STK17B could potentially increase the clinical response rates in tumors with a low mutational burden. However, despite the compelling T cell phenotype of *STK17B* knockout mice, it remains unclear whether these observations are linked to its kinase activity, or if potential scaffolding functions of STK17B could be contributing as well. Hence, we sought to determine whether inhibition of STK17B with a small molecule in peripheral T cells would lead to antitumor effects.

At the time of this work, no small molecule inhibitors were available in the public domain to validate STK17B as a cancer immunotherapy target, nor assays to enable investigation of STK17B, such as biochemical and cellular target engagement assays, or pharmacokinetic (PK)/pharmacodynamic (PD) models. Therefore, to evaluate STK17B as a potential target, we developed appropriate assays and optimized tool compounds derived from Blueprint Medicines Corporation's proprietary kinase inhibitor library. Here, we report the development and validation of a series of highly potent and selective STK17B tool compounds, and the results of our target validation work.

## Methods

### Enzyme assay

Sulfonamido-oxine (Sox) chromophore peptide substrates were purchased from AssayQuant Technologies (Marlboro, MA, USA). Enzyme inhibition was studied as a function of inhibitor concentration using a Sox-based fluorescence assay that allows real-time measurement of enzyme activity (10–12). In this assay, kinase activity is measured by an increase in fluorescence as a result of phosphorylation of a Sox-labeled peptide substrate. Briefly, Substrate Mix containing 2× (20 µM). Sox-labeled substrate was prepared in assay buffer, which comprised 50 mM HEPES (N-2-hydroxyethylpiperazine-N’-2-ethanesulfonic acid), pH 7.5, 10 mM MgCl_2_, 50 mM KCl, 0.5 mM EGTA, 0.002% NP-40, and 0.5 mM fresh TCEP (tris(2-carboxyethyl)phosphine). Next, 5 μL of Substrate Mix was added to the assay plate (Greiner non-binding low volume black plate), and 100 nL of compound serial dilution in dimethyl sulfoxide (DMSO) was added to each well by Mosquito (SPT Labtech). Reactions were initiated with the addition of 5 μL of Enzyme+ATP (adenosine 5′-triphosphate) Mix containing 5 nM GST-DRAK2 (BPS Biosciences) and 2 mM ATP in assay buffer. The final assay contained 2.5 nM GST-DRAK2 (or 10 nM GST-DRAK1, Carna Biosciences), 10 μM Sox-labeled substrate (AssayQuant), and 1 mM ATP in assay buffer.

Fluorescence intensity readings were measured using a Synergy NEO plate reader (Biotek). Half maximal inhibitory concentration (IC_50_) values for compounds against STK17A and STK17B were subsequently calculated using four parameter logistic equation.

### NanoBRET cellular target engagement assay

A Nano-Luc luciferase-based bioluminescence resonance energy transfer (BRET) target engagement assay was used to determine cellular target engagement of selected compounds in human Flp-In T-REx-293 cells. Flp-In T-REx-293/STK17A-C-NanoLuc or Flp-In T-REx-293/STK17B-C-NanoLuc cells were seeded at a density of 5×10^3^ cells per well in 3x 384-well white plates using a multidrop dispenser and incubated overnight in media with 100 ng/ml doxycycline. The next day, media was aspirated using a microplate automatic washer and the remaining media were removed by patting the plate on a paper towel. Next, 27 µL of pre-warmed Opti-MEM/Probe 1 and 3 µL of diluted 100% DMSO or 10x respective test compounds were added per well.

Cells, Probe 1, and compounds were mixed in an orbital shaker for 3 min at room temperature (RT) and then incubated at 37°C, 5% CO_2_ in a humidified incubator for two hours. Plates were removed from the incubator, allowed to cool to room temperature for 15 min and then 15 µL of 3× complete NanoBRET Nano-Glo Substrate/Inhibitor was added per well. Plates were mixed in an orbital shaker for 3 min at room temperature and BRET measurements (NanoBRET Dual Emission Label set #2100-8530, Perkin Elmer) were determined in an EnVision instrument (Perkin Elmer) within 10 minutes of substrate addition.

### *In vitro* T cell interleukin-2 (IL-2) and OT-1 proliferation assays

#### IL-2 functional assay

C57BL/6 mouse spleen was harvested, and T cells were purified using EasySep Mouse T Cell Isolation Kit (Stemcell Technologies) according to the manufacturer’s protocol. T cells were incubated with different concentrations of STK17B inhibitors or DMSO control. 50K cells/well were plated in triplicate on anti-cluster of differentiation 3 (anti-CD3) antibody-coated 96-well plates (1.5 ug/mL clone 145-2C11 in 0.1 M carbonate bicarbonate buffer, pH 9, coated at 4°C overnight) with 2 ug/mL anti-cluster of differentiation 28 (anti-CD28) antibody (Clone 37.51, BD Pharmingen) added to T cell culture media (RPMI1640 with 10% fetal bovine serum (FBS) supplemented with 1 mM sodium pyruvate, 2 mM glutamine, 10 mM HEPES buffer solution and 50 µM β-mercaptoethanol). After incubation for 24 hours at 37°C, 5% CO_2_, plates were centrifuged at 1000 rpm for 5 min. IL-2 secretion in the supernatant was analyzed by R&D system IL-2 DuoSet ELISA (13).

#### OT-1 proliferation assay

The cluster of differentiation 8 (CD8)-positive T cells were purified from OT-1 mouse spleens using an EasySep Mouse CD8+ T cell Isolation Kit (Stemcell Technologies) according to the manufacturer’s protocol. Leftover splenocytes were treated with 50 ug/mL mitomycin C for 30 minutes at 37°C, washed four times with culture medium and then used as antigen presenting cells in the assay in the presence of 2 ng/mL SIINFEKL peptide derived from ovalbumin (AnaSpec). 25K T cells and 25K splenocytes per well were incubated in triplicate with different concentrations of STK17B inhibitors at 37°C, 5% CO_2_ for 96 hours. Cell proliferation and viability at the end of the assay were determined with CellTiter Glo^®^ assay (Promega) according to the manufacturer’s protocol and luminescence was measured on an EnVision instrument (Perkin Elmer).

### Human T cell activation assay

MV3 melanoma cancer cell line (in-house) was seeded in 100 µL assay medium (RPMI1640+ Glutamax with 10% FBS) into a 96-well flat bottom plate (Costar) at a concentration of 0.5×10^6^ cells/mL. T cells were isolated from freshly thawed human peripheral blood mononuclear cells (Stem Cell Technology) using the human Pan T Cell Isolation Kit (Miltenyi) following the manufacturer’s manual. Next, T cells were adjusted to a concentration of 2×10^6^ cells/mL in assay medium and 100 µL was added to MV3 cells, resulting in a target to effector cell ratio of 1:4. As a stimulant, 0.1 nM T cell-bispecific antibody (MCSP-TCB) were added, cross-linking cancer cells and T cells.

As a control, an antibody containing only a functional anti-CD3 arm, but no functional antigen-binding arms was used (DP47). TCB details are described in the US patent application 2014/0242079 (14). The compound of interest was added, and samples were incubated for 96 hours at 37°C and 5% CO_2_. After 96 hours, the supernatants were harvested and frozen at –20°C until the measurement of cytokines.

Interleukin-2 concentrations were assessed using Cytometric Bead Array Flex sets (BD). Next, 10 µL samples were combined with 10 µL Capture Bead Mix (Capture beads were diluted 1:50 in the corresponding diluent) and incubated for one hour at room temperature on a shaker (350 rpm). Afterward, 10 µL of Detection Reagent Mix (from the BD cytometric bead array kit) was added and the mixture was incubated for another two hours at room temperature on a shaker (350 rpm). Samples were washed with 150 µL Wash Buffer and resuspended in 40 µL Wash Buffer. Data acquisition was performed using an iQue flow cytometer (Sartorius).

### Mass spectrometry

A discovery phosphoproteomics experiment was performed using mass spectrometry to identify STK17B substrates. Please refer to the **Supplementary Materials** for more details.

### Peripheral blood phospho-myosin light chain 2 flow-cytometry assay

C57BL/6 mice were dosed with STK17B inhibitor compound. Blood was obtained by submandibular bleeding into heparin tubes. 50 µL of blood were transferred into a 96-well V-bottom plate (Thermo Fisher, Waltham, Massachusetts, USA), stained with anti-CD3 (1:50, BioLegend, Clone 17A2) and 1:100 Zombie Aqua (BioLegend 423102) for 30 min, washed twice with stain buffer (BioLegend Cat #42201), resuspended in Cytofix/Cytoperm Solution (BD Cat#554714), and incubated for 20 min at 4°C in the dark. Cells were washed three times with 100 µL/well Perm/Wash Buffer (BD Cat#554714) and centrifuged at 400×g for 5 min at 4°C. Cells were then incubated with anti-phospho-myosin light chain 2 (pMLC2) S19 antibody (Cell Signaling Technology Cat# CST 3675, Lot #5) diluted 1:400 in Perm/Wash buffer for one hour on ice in the dark.

Cells were subsequently washed three times as above and then incubated with Rat anti–mouse-IgG1 antibody (BioLegend 406618) diluted in Perm/Wash Buffer 1:800 for one hour on ice in the dark. Next, cells were washed three times with Perm/Wash buffer as above, then resuspended in 100 µL Stain buffer and transferred to a 96-well MultiScreen-MESH Filter plate (Millipore) and centrifuged at 100×g for 1 min at RT. Data were acquired using an Attune Flow Cytometer calibrated with Attune Performance Tracking Beads (Thermo Fisher, Waltham, Massachusetts, USA) and analyzed using FlowJo software (BD Biosciences, Franklin Lakes, New Jersey, USA).

### Effect of STK17B inhibition on SIINFEKL peptide immunization

OT-1 mice (C57BL/6-Tg[TCRaTCRb]1100Mjb/J) six to eight weeks of age were immunized with 10 or 3 µg SIINFEKL peptide (AnaSpec) in Montanide ISA 70 Vg adjuvants (SEPPIC) by subcutaneous injection. Animals were dosed with 200 mg/kg BLU7482 in 30% 2-hydroxypropyl-β-cyclodextrin (HP-β-CD) in 100 mM citrate, pH 3, by oral gavage.

Blood was collected by submandibular bleeding for serum cytokine monitoring at 24 hours post dose and animals were sacrificed at 48 hours post dose. Draining lymph nodes were harvested and single cell suspensions were obtained by passing through a Falcon 70 µm filter, subsequently stained for immunophenotyping, and analyzed on a BD Fortessa flow cytometer. Data was analyzed using FlowJo software (BD Biosciences, Franklin Lakes, New Jersey, USA)

Antibodies from BioLegend were used for flow cytometry for the detection of CD3 (Clone 145-2C11 Cat# 100321) 1:100 dilution, CD8 (Clone 53-6.7 Cat # 100732) 1:100 dilution, IL-2 receptor alpha chain (CD25) (Clone PC61, Cat #102049) 1:100 dilution and the cluster of differentiation 69 (CD69) (Clone H1.2F3, Cat #104512) 1:100 dilution. Serum cytokine concentrations were determined with a Meso Scale Discovery assay (V-Plex pro-inflammatory panel SULFO-TAG antibodies for IL-2, interferon-γ, tumor necrosis factor α), according to the manufacturer’s protocol.

### OT-I adoptive T cell transfer experiments

T cells were isolated from CD45.2+ OT-I TCR transgenic splenocytes. OT-I T cells were transplanted in CD45.1+ mice via tail vein injections (3 million cells per recipient mouse). One day after the adoptive OT-I T cell transfer, mice were treated with different doses of BLU0556. To isolate OT-I T cells, TCR transgenic OT-I mice were sacrificed, spleens were isolated, and splenocyte suspensions were prepared with the help of cell strainers. One hour after compound treatment, recipient mice were vaccinated with ovalbumin (OVA) peptides of different sequence/affinity to major histocompatibility complex class I (MHC-I) (high affinity: SIINFEKL & low affinity: SIIQFELK) or as a control with adjuvants only (Montanide ISA 720, Seppic).

After immunization, mice were treated for the next two days with different doses of BLU0556. Three days after treatment started, mice were sacrificed and draining inguinal lymph nodes were isolated. OT-I T cell frequencies were determined by flow cytometry on BD LSR Fortessa flow cytometer with staining for congenic markers and T cell markers (CD45.1 (Clone A20, Biolegend Cat #110708) CD45.2 (Clone 104, Biolegend Cat #109826), CD3 (Clone 145-2C11, Biolegend Cat# 100321), CD8 (Clone 53-6.7, Biolegend Cat #100732), CD4 (Clone GK1.5, Biolegend Cat #100428), 1:100 respectively). Data were analyzed using FlowJo software (BD Biosciences, Franklin Lakes, New Jersey, USA).

### Effect of BLU7482 on MCA205 and MC38 tumor growth in combination with anti–PD-L1

Female C57BL/6 mice, eight weeks of age, were implanted on the right flank subcutaneously with 10^6^ MCA205 cells or 10^6^ MC38 cells in 0.1 mL of a 1:1 mixture of medium and Matrigel. When tumors reached over 50 mm^3^ for MCA205 cells or 50–80 mm^3^ for MC38 cells on average, mice were randomized into three groups (n = 10/group) and dosed either with anti–PD-L1 antibody (15) (Roche, Basel, Switzerland) alone (10 mg/kg intraperitoneal [i.p.] day 1, then 5 mg/kg i.p. every three days) or in combination with 200 mg/kg BLU7482 in 30% (w/w) HP-β-CD in 100 mM citrate buffer, pH 3 each day.

Tumor size was assessed daily utilizing automatic electronic calipers. The study was terminated when the vehicle animal group reached 1500 mm^3^. Terminal PK was taken by micro-sampling at 1, 2, 4, 8, and 24 hours after the last dose and analyzed via liquid chromatography-mass spectrometry at an external contract research organization (Charles River).

### Ethics approval

Animal experiments were performed with approval from the Institutional Animal Care and Use Committee and according to committed guidelines (GV-Solas; Felasa; TierschG) with the approved protocol ROB-55.2.2532.Vet_03-20-30.

## Results

### Identification and development of suitable *in vivo* kinase inhibitor compounds

**Figure 1:**
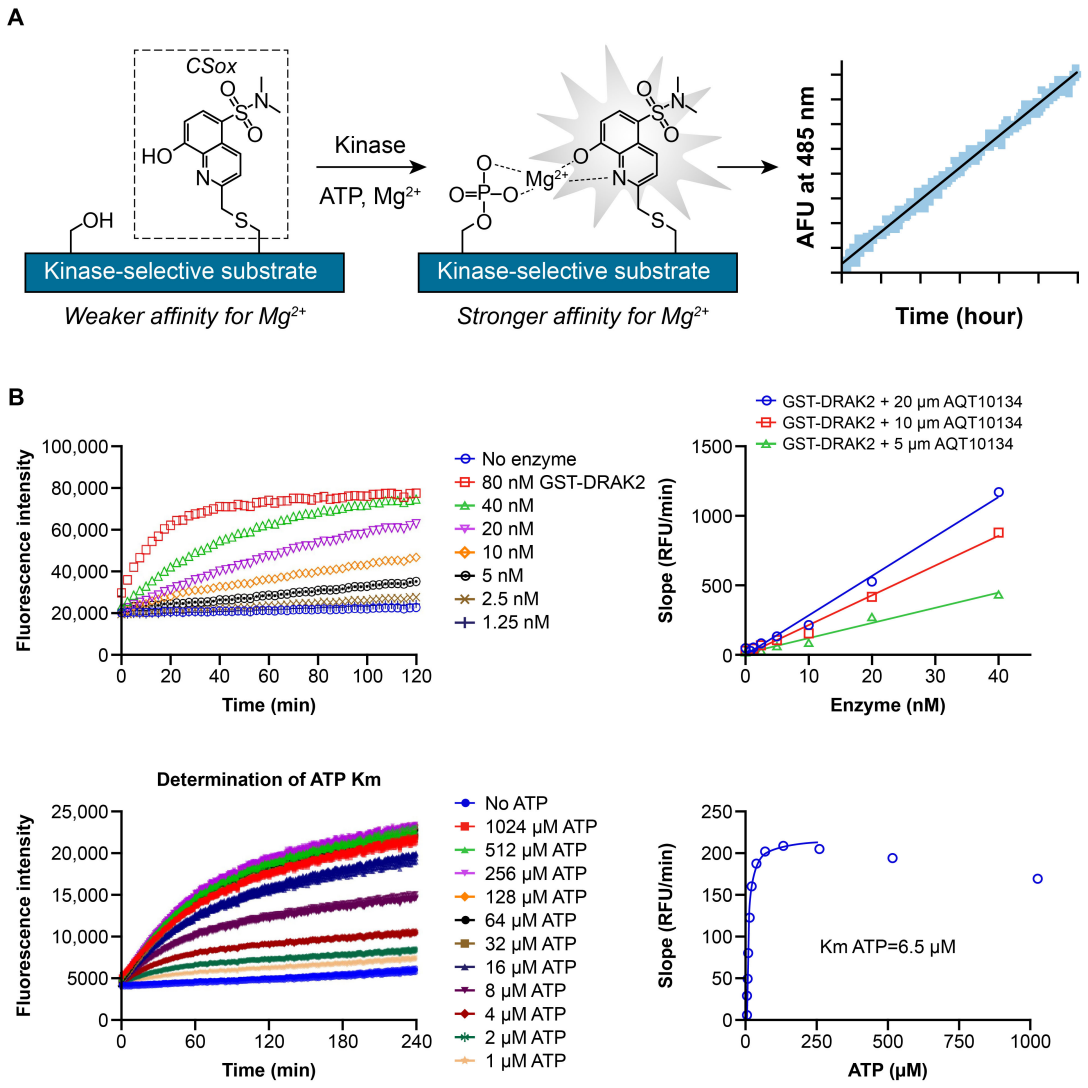
PhosphoSens assay development **(A)**, enzyme titration and ATP Km determination **(B)**. **(A)** Step 1: A library of ~1000 serine/threonine peptides of ~18 amino acid residues (based on known phosphorylation sites) was screened against STK17B kinase to identify the best substrate peptide (Ac-KAKTTKKRPQRATSN-C(Sox)-FS-amide). Step 2: Each amino acid position was mutated and evaluated. Step 3: Single mutations identified in the second step are combined in a matrix manner and again screened against STK17B kinase. The final optimized substrate peptide for STK17B has the sequence Ac-KKKKVKKRPQRADSD-C(Sox)-FA-amide. **(B)** With the optimized substrate peptide, enzyme titration of STK17B (labeled as GST-DRAK2) from SignalChem was performed from 80 nM top concentration with 2-fold dilution down (0, 1.25, 2.5, 5, 10, 20, 40, 80 nM) in the presence of 1 mM ATP. ATP Km was determined by measuring initial velocity (fluorescence intensity versus time) at 1-1024 µM ATP. The initial velocity was plotted against ATP concentration and fitted with the Michaelis−Menten equation to give Km of 6.5 µM for STK17B. ATP, adenosine triphosphate; DRAK2, DAP kinase-related apoptosis-inducing protein kinase 2; GST, glutathione S-transferase; RFU, relative fluorescence units; Sox, Sulfonamido-oxine; STK, serine/threonine kinase.

To enable measurement of compound inhibition against STK17B and its paralog, STK17A, enzymatic activity assays were developed using PhosphoSens^®^ assay technology (AssayQuant Technologies) based on peptide incorporating cysteine-Sox fluorophore (**Figure 1A**). Stepwise peptide substrate identification and optimization were employed to identify an optimal substrate peptide for STK17B (AssayQuant). In the 1^st^ step, a library of ~1000 serine/threonine peptides of ~18 amino acid residues were screened against STK17B kinase to identify the best substrate peptide. In the 2^nd^ step, each amino acid position was mutated and evaluated again. Finally in the 3^rd^ step, single mutations identified in the 2^nd^ step were combined in a matrix manner and screened against STK17B kinase. The optimized substrate peptide for STK17B has the sequence Ac-KKKKVKKRPQRADSD-C(Sox)-FA-amide and gives high level of phosphorylation by both STK17B and STK17A. Using this substrate peptide, enzyme titration and ATP Km determination for STK17B were performed and data are shown in **Figure 1B**.

Blueprint Medicines Corporation has a proprietary kinase inhibitor library in which every compound has been tested against the scanMAX KINOMEscan panel (Eurofins). In this panel, compound is tested at a single concentration for its ability to competitively bind to barcoded kinases and to displace immobilized probe. Based on these data, compounds were identified from the library that not only showed potent binding to the target STK17B (confirmed by kinase assay), but also had limited number of off-target kinases. To quantify the kinome promiscuity of any inhibitor, we use a selectivity score S(10) at 3 µM which measures the percentage of wild type kinases within the scanMAX panel that’s inhibited more than 90% at the compound concentration of 3 µM, i.e., an S(10) score of 0.1 would mean 10% of 400 kinases in the panel, or 40, are inhibited by 90% by a given compound at the specified concentration. Our initial library hits have STK17B potency (enzyme IC_50_ values) under 200 nM and S(10) score less than 0.1.

In human but not in mouse, STK17B has a closely related homologue STK17A (also called DRAK1) which shares 60% amino acid identity. In order to interrogate the function of STK17B in human T cell-based functional assays, we sought to optimize compounds that selectively inhibit STK17B over STK17A. This optimization work is enabled by STK17A/B enzyme and NanoBRET target engagement assays, which correlate well with each other (**Supplementary Figure S1**). Broad kinome selectivity is monitored by KINOMEscan profiling. Compounds were optimized through iterative design, make, and test cycles. This led to a potent (enzyme IC_50_ value of 12.5 nM) and selective (70-fold selective over STK17A, S(10) score of 0.067) *in vitro* tool compound BLU4039, as shown in **Supplementary Table S1**. Having this *in vitro* tool compound allowed us to perform phosphoproteomics profiling experiments (described in section below with heading “Modulation of pMLC2 *in vitro* and *in vivo*”) to identify physiological substrate of STK17B. We also identified and optimized, to a limited extent, STK17A-selective tool compounds, BLU9887 and BLU7120 for comparison (**Supplementary Table S1**).

**Figure 2:**
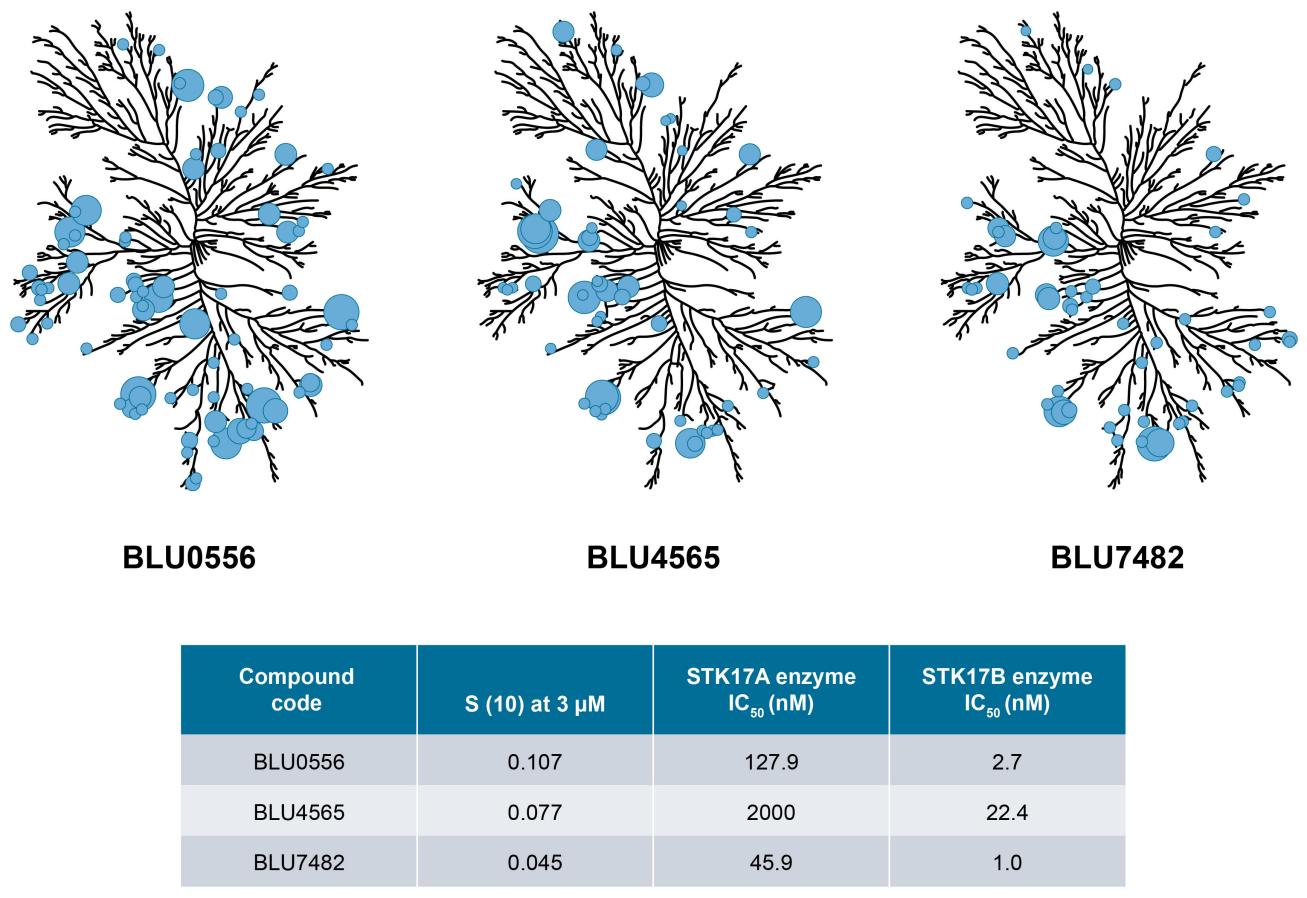
KINOMEscan trees for selected compounds. Kinome selectivity of tool compounds is measured against >400 human kinases in the scanMAX KINOMEscan^®^ panel. Compounds were tested at 3 μM final concentration. Circles indicate the major kinase hits and their size is inversely proportional to estimated binding affinity. The enzyme inhibition IC_50_ values for selected compounds against STK17A and STK17B measured at 1 mM ATP are also shown. Kinome illustration was reproduced courtesy of Cell Signaling Technology, Inc. (www.cellsignal.com) (CSTI). The foregoing website is maintained by CSTI and Blueprint Medicines Corporation is not responsible for its content.

Subsequent chemistry optimization focused on ADME and pharmacokinetics properties, in addition to further improving potency and selectivity, ultimately leading to three advanced compounds, BLU0556, BLU4565, and BLU7482. These 3 lead compounds showed 40~90-fold selectivity by enzyme assays, and 50~150-fold selectivity by NanoBRET assay, as described in **Supplementary Table S1**. To demonstrate their kinome selectivity, the KINOMEscan trees for these 3 compounds are shown in **Figure 2**. Their physicochemical properties and *in vivo* PK parameters are shown in **Table 1**.

**Table 1:** Properties of selected compounds with acceptable *in vivo* profiles.

Compound properties	BLU4565	BLU0556	BLU7482
**MW**	461.5	413.5	420.5
**cLogP, cLogD**	4.6, 4.6	4.3, 4.3	3.6, 2.6
**TPSA**	97	92	97
**FaSSIF solubility (µM)**	100	32	100
**Mouse IV CL (mL/min/kg) (CL_u_)**	14.7 (381)	26 (>3000)	71.3 (552)
**Mouse bioavailability (%F)**	100	>50	77
**S(10) score**	0.077	0.107	0.045

CL, clearance; cLogP; partition coefficient; cLogD, distribution coefficient; FaSSIF, fasted state simulated intestinal fluid; IV, intravenous; MW, molecular weight; S(10) score, selectivity score defined by the fraction of kinases with PoC <10% when measured against KINOMEscan panel at 3 µM compound concentration; TPSA, total polar surface area.

### Effects of STK17B inhibitors on IL-2 production and downstream TCR signaling *in vitro*

**Figure 3:**
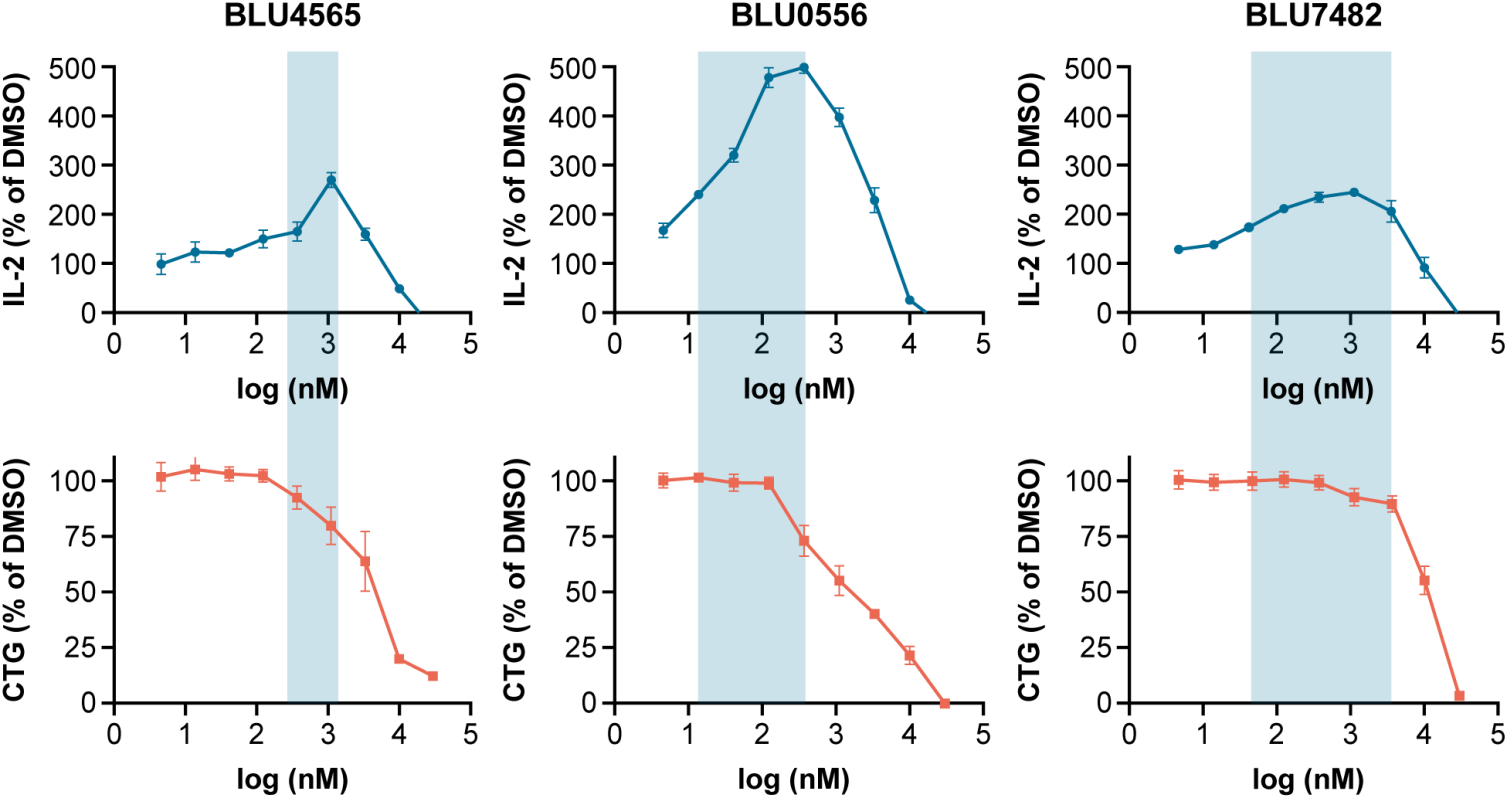
BLU4565, BLU0556, and BLU7482 modulation of IL-2 release. C57BL/6 splenocytes were incubated with plate bound anti-CD3 (Clone 145-2C11, 1 µg/mL) in the presence of DMSO or different concentrations of STK17B inhibitor in 96-well in triplicate. After 24 hours, IL-2 secretion was measured in supernatants to assess the ability of compounds to enhance T cell activation (top panel). In parallel, OT-1 splenocytes were incubated with 1 µg/mL SIINFEKL peptide in the presence of DMSO or different concentrations of STK17B inhibitors and cultured for 96 hours. Cell-titer glow measurements assessed any off-target activity that inhibits proliferation (bottom panel). The aim was to identify potent compounds with a wide window between IL-2 induction and inhibition of proliferation – with BLU7482 reaching a 163× window. Representative of n=3 experiments is shown. CD, cluster of differentiation; CTG, CellTiter-Glo; DMSO, dimethyl sulfoxide; EC_50_, half maximal effective concentration; IC_50_, half maximal inhibitory concentration; IL-2, interleukin-2.

Compounds were tested in a mouse *in vitro* T cell activation assay (with plate-bound anti-CD3 antibody) to assess their capability to enhance IL-2 secretion. Potential off-target effects on T cell proliferation and general T cell viability were determined in a cell proliferation assay where OT-1 T cells were stimulated with ovalbumin-derived SIINFEKL peptide in the presence of antigen presenting cells (splenocytes). Of investigated compounds, BLU7482 demonstrated the best half maximal effective concentration (EC_50_) for IL-2 induction to OT-1 proliferation IC_50_ window (163×) (**Figure 3**, **Supplementary Table S2**), ensuring that any residual off-target effects of the inhibitor would not interfere with T cell viability nor proliferation downstream of T cell activation. Therefore, this compound is ideal for our *in vivo* proof-of-concept experiments since it showed the desired *in vitro* effects and favorable PK.

A critical component of T cell signaling pathway downstream of T cell receptor ligation is intracellular calcium flux. STK17B has been implicated in attenuating calcium flux in T cells downstream of PLCγ and PKD, by negatively regulating ORAI1 (calcium release-activated calcium modulator) (5). Our *in vitro* tool compound BLU4039 was evaluated in a FLIPR assay and showed dose-dependent enhanced calcium flux upon TCR activation in human T cells (**Supplementary Figure S2A**). This observed increase in calcium signaling is also consistent with the finding that STK17B inhibition allows T cells to be activated with suboptimal antigens. STK17A- and STK17B-selective compounds (described in **Supplementary Table S1**) were also tested in a human T cell assay to understand whether STK17A plays any role in human T cells. This this end, human donor-derived T cells were co-cultured with melanoma cells and stimulated with a melanoma targeting T cell bispecific antibody (MCSP-TCB) in presence of STK17A- or STK17B-selective compounds. Only STK17B inhibitors were found to induce production of IL-2 cytokine in human T cells (**Supplementary Figure S2B**), indicating the desired T cell-sensitizing activity is restricted to STK17B, but not its paralog STK17A in this context. After confirming in human T cells, that STK17B rather than STK17A is responsible for the same phenotypic changes that are observed in STK17B-deficient mouse T cells, we focused on developing STK17B-selective compounds, because there was no advantage to maintain STK17A activity and avoiding it may minimize any STK17A-related potential safety risk, given that the full function of STK17A is unknown. Finally, to rule out that our STK17B inhibitors modulate T cell function by off-target effects that are unrelated to their STK17B inhibitory function, the effect of compound BLU4039 on T cell proliferation was assessed in T cells from STK17B-knockout mice compared to wild type (WT) littermate controls. BLU4039 only enhanced T cell proliferation in the presence of functional copy of STK17B gene, while STK17B-deficient T cells showed higher proliferation than WT controls as expected, and no additional effect was observed upon compound treatment (**Supplementary Figure S3**). Together, these data demonstrate that our STK17B inhibitors exert their activity on T cells (enhanced calcium flux, increased IL-2 secretion, and enhanced proliferation) in a selective manner solely through inhibition of STK17B kinase activity.

### Effects of STK17B inhibitors *in vivo*

#### Modulation of pMLC2 *in vitro* and *in vivo*

In order to identify a pharmacodynamic biomarker that could be used to monitor STK17B inhibition *in vivo*, we employed mass spectrometry-based phosphoproteomics technology to measure on a global scale, phosphorylation changes upon treatment of mouse T cells with BLU4039. Serine 19 on MLC2 was identified as the most significantly modulated STK17B substrate (**Supplementary Figure S4**). Because ROCK1/2 kinases have also been reported to phosphorylate serine 19 on MLC2, we also tested the impact of ROCK1/2 kinase inhibition in T cells. In this experiment, the ROCK1/2 inhibitor GSK429286A was added to mouse T cells along with the STK17B inhibitor BLU4565. Further reduction of pMLC2 median fluorescence intensity signal was minimal compared with BLU4565 treatment alone, demonstrating that STK17B kinase activity accounts for the majority of the phosphorylation activity at this residue (**Supplementary Figure S5A**). Dose response curves allowed the determination of IC_50_ values for BLU0556 and BLU4565, which were 1120 and 3073 nM, respectively (**Supplementary Figure S5B**). STK17B inhibition in NanoBRET assay generally correlated with pMLC2 in mouse T cells (**Supplementary Figure S5C**), with shift in IC_50_ values likely attributable to serum protein binding.

**Figure 4:**
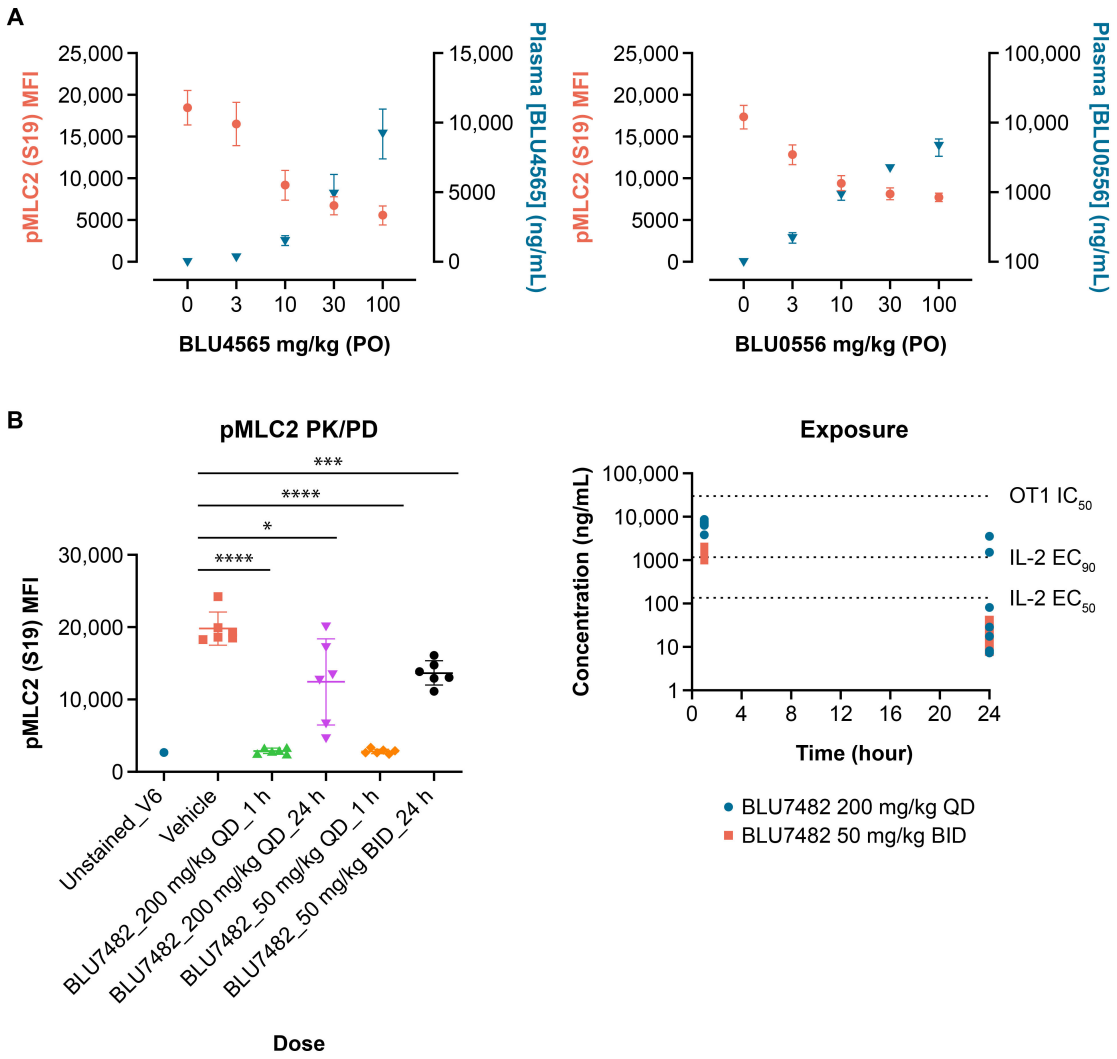
*In vivo* pMLC2 modulation. **(A)** Female, 8-week-old C57/BL6 mice (n = 8 per group) were dosed with indicated amounts of BLU4565 or BLU0556, respectively. Submandibular bleeds were taken one hour after dosing, and samples were analyzed by flow cytometry. Plasma samples were also taken to measure compound exposure. For pMLC2 flow cytometry, red blood cells were lysed, lymphocytes were stained with anti-CD3, and intracellular staining for pMLC2 was performed. **(B)** C57BL/6 mice (n = 6 per group) were dosed with BLU7482 (200 mg/kg QD or 50 mg/kg BID). Whole blood was collected through submandibular vein at 1 or 24 hours post treatment, followed by immunostaining and FACS analysis. Plasma samples were taken at 1 and 24 hours to determine compound exposure. Representative of n=3 experiments is shown. *P=0.0178, ***P=0.0003, ****P<0.001. BID, twice a day; CD, cluster of differentiation; EC_50_, half maximal effective concentration; IL-2, interleukin-2; MFI, mean fluorescence intensity; PD, pharmacodynamics; PK, pharmacokinetics; pMLC2, phospho-myosin light chain 2; P.O., orally; QD, daily.

Three lead compounds, BLU0556, BLU4565, and BLU7482, with good potency, selectivity, and desired PK properties were described earlier as shown in **Figure 2** and **Table 1**. In subsequent animal studies, BLU4565 and BLU0556 were found to modulate pMLC2 levels in a dose-dependent manner, confirming target engagement (**Figure 4A**). In a separate experiment, BLU7482 modulated the pMLC2 levels *in vivo* in a time-dependent manner (**Figure 4B**). Dosing of BLU7482 either at 200 mg/kg QD or 50 mg/kg twice daily (BID) was able to continuously cover ≥50% inhibition of pMLC2 over 24 hours. The PK profile of this compound was projected to cover the EC_50_ value of IL-2 induction while staying lower than the IC_50_ value of OT-1 proliferation assay, suggesting a good window between enhanced T cell activation and any negative impact on T cell proliferation that could be due to off-target activities.

#### Effect of STK17B inhibitor on T cell activation, IL-2 secretion, and T cell proliferation after suboptimal antigen receptor stimulation *in vivo*

**Figure 5:**
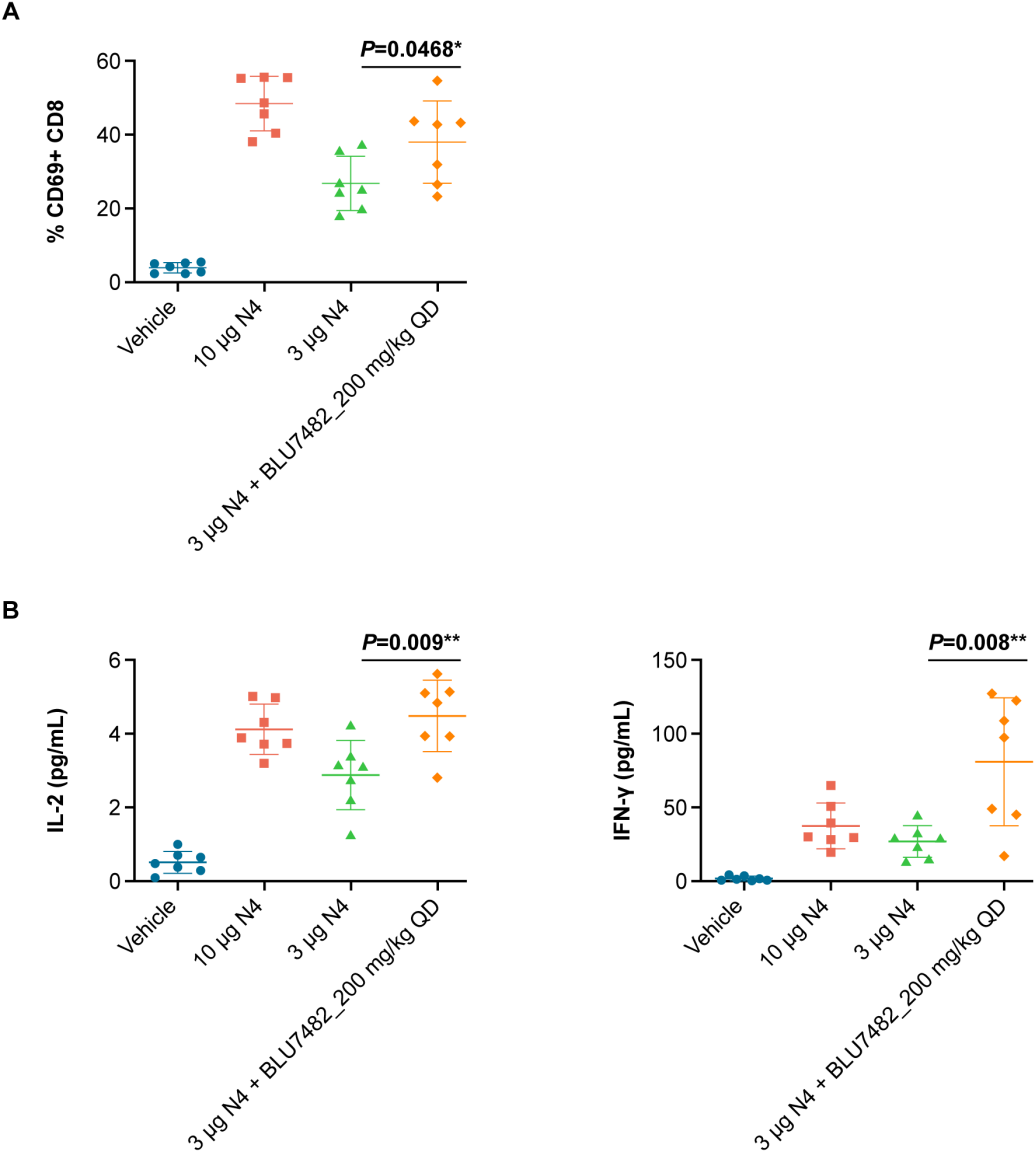
Effect of STK17B inhibition on T cell activation *in vivo*. **(A)** 7 female OT-1 mice (8 weeks of age) were immunized with 10 µg or 3 µg SIINFEKL peptide and dosed with or BLU7482 (200 mg/kg daily, orally in solution formulation (30% HP-β-CD in 100 mM citrate; pH 3). Lymph nodes were collected, single cell suspension was generated by passing through 70 µm mesh filter, and T cell activation was assessed by flow cytometry measuring the expression level of early T cell activation marker CD69. **(B)** Serum was collected through submandibular vein at 24 hours post treatment, followed by mouse cytokine assay for IL-2 and IFN-γ (Meso Scale Discovery). Representative of n=3 experiments is shown. Statistics: non-parametric T test (GraphPad PRISM). CD, cluster of differentiation; HP-β-CD, 2-hydroxypropyl-β-cyclodextrin; IL-2, interleukin-2; IFN-γ, interferon‐γ; QD, daily; STK, serine/threonine kinase.

BLU7482 was identified as a highly potent and selective STK17B inhibitor with favorable pharmacokinetics. To determine the effect of STK17B inhibition on T cell activation *in vivo*, OT-1 mice were immunized with either optimal or suboptimal concentrations of ovalbumin-derived SIINFEKL peptide. STK17B inhibition was found to enhance *in vivo* T cell activation under suboptimal immunization conditions when OT-1 mice were immunized with a low dose (3 µg) of SIINFEKL peptide and treated with BLU7482, as evidenced by both upregulation of early activation marker CD69 (**Figure 5A**) and enhanced secretion of cytokines (**Figure 5B**).

Treatment with STK17B inhibitor BLU0556 also increased T cell proliferation *in vivo*. In an adoptive transfer experiment, OT-I T cell numbers (CD45.2+ cells) were increased by BLU0556 treatment (**Supplementary Figure S6**). T cell numbers also increased after BLU0556 treatment in suboptimal stimulation conditions, utilizing an altered ovalbumin peptide (SIIQFEKL), to the same levels of stimulation with optimal SIINFEKL peptide (**Supplementary Figure S6**).

#### Antitumor activity of STK17B inhibitor BLU7482 in syngeneic mouse models

**Figure 6:**
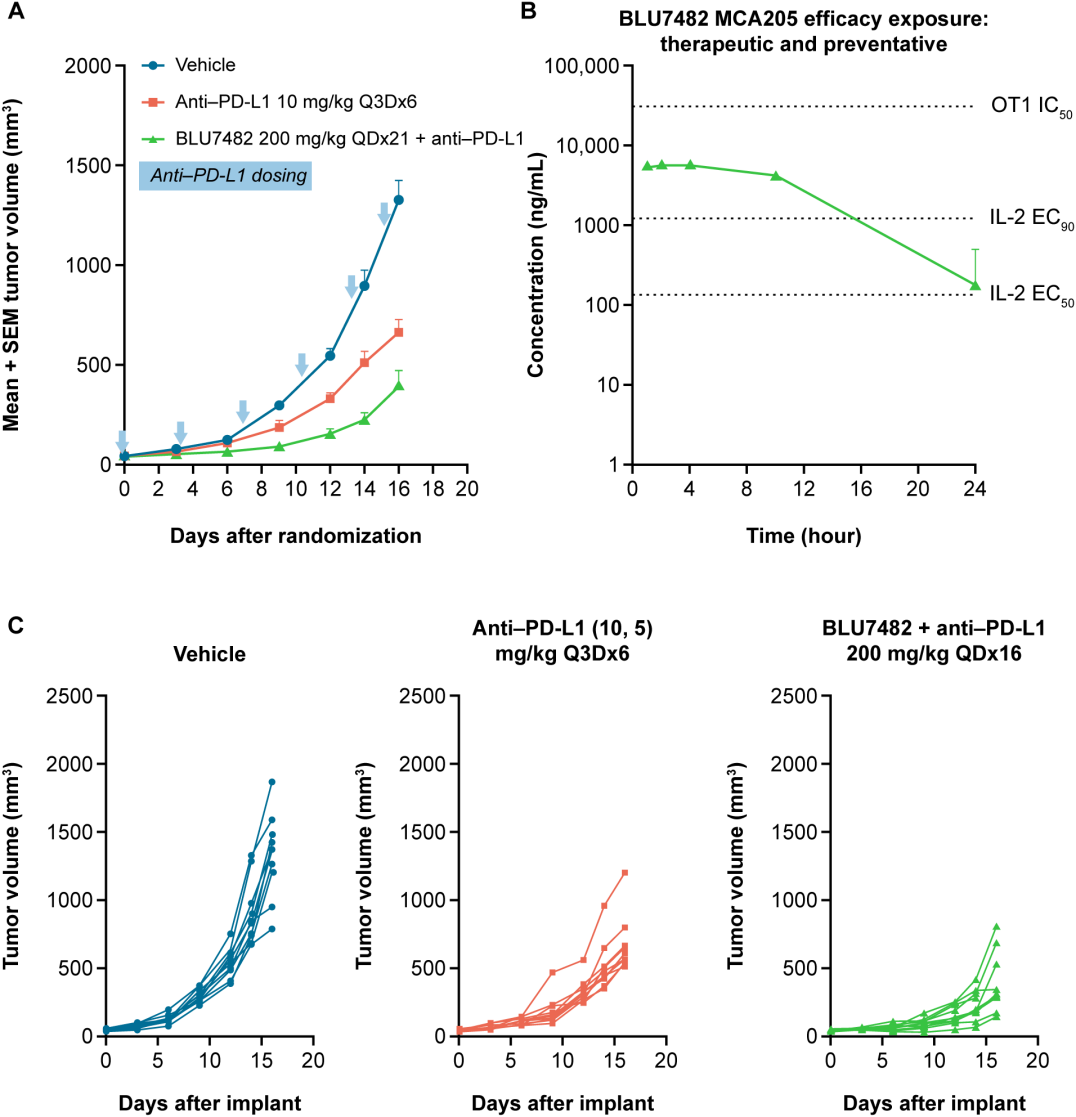
*In vivo* activity of BLU7482 in MCA205 mouse model on tumor volume **(A)**, efficacy exposure **(B)**, and tumor volume in individual mice **(C)**. **(A)** Tumor growth curves of 8-week-old, female C57/BL6 MCA205-bearing mice (n = 10 per group) after treatment with either anti–PD-L1 monoclonal antibody alone (10 mg/kg i.p. day of treatment start and then 5 mg/kg every three days until study end [red line]), or in combination with 200 mg/kg QD of BLU7482 (green line). Vehicle group (blue line) had no treatment. The blue arrows denote anti–PD-L1 dosing. The zero time-point indicates the initiation of treatment. **(B)** Concentration−time curve of BLU7482. **(C)** Tumor growth curves of individual mice: left panel: vehicle (no treatment); middle panel: treated with anti–PD-L1 monoclonal antibody alone; right panel: treated with anti–PD-L1 and 200 mg/kg QD of BLU7482. EC_50_, half maximal effective concentration; EC_90_, 90% maximal effective concentration; IC_50_, half maximal inhibitory concentration; IL-2, interleukin-2; i.p., intraperitoneal; anti–PD-L1, anti-programmed death-ligand 1; Q3D, every three days; QD, daily, SEM, standard error of the mean.

In the immunogenic MCA205 mouse sarcoma model, dosing of 200 mg/kg BLU7482 daily led to enhanced antitumor activity in combination with anti–PD-L1 compared with anti–PD-L1 alone (**Figure 6**). However, BLU7482 exhibited minimal effect either as monotherapy or when combined with anti–PD-L1 treatment in another syngeneic tumor model (MC38) (**Supplementary Figure S7**). These findings are discussed in the section below.

## Discussion

This work investigated the serine/threonine protein kinase STK17B as a potential cancer immunotherapy target. A series of potent and selective inhibitor compounds were developed based on starting points from Blueprint Medicines Corporation’s proprietary kinase inhibitor library. Once suitable compounds with desired cellular potency and selectivity against both closely related paralog STK17A and broad kinome were generated, their ability to enhance T cell activation was demonstrated *in vitro* in human and mouse T cell activation assays. Using an *in vitro* tool compound and mouse T cells, the team subsequently performed phosphoproteomics study and identified residue serine 19 of MLC2 as a substrate of STK17B. Subsequent screening and optimization using pMLC2 by flow cytometry as *in vivo* PD readout led to identification of a highly optimized STK17B inhibitor, BLU7482, with suitable *in vivo* exposure. Finally, the antitumor activity of tool compound BLU7482 was evaluated in multiple mouse tumor models in combination with anti-PD-L1 antibody.

STK17B inhibition *in vivo* led to enhanced priming of naïve T cells as evidenced by up-regulation of early activation marker CD69, greater secretion of IL-2 and interferon-γ, as well as expansion of antigen-specific T cells in the periphery. The antitumor activity of BLU7482 was assessed in two mouse models. In the highly immunogenic MCA205 model, BLU7482 demonstrated enhanced activity when combined with anti–PD-L1 vs anti–PD-L1 alone (**Figure 6**). This boost in the antitumor activity of anti-PD-L1 antibody is notable, given that BLU4782 as single agent shows very moderate activity in a prophylactic setting (**Supplementary Figure S8**). However, when investigated in an additional syngeneic tumor model MC38, BLU7482 showed minimal to no effect, either as monotherapy or when combined with anti–PD-L1 treatment (**Supplementary Figure S7**). While these studies were done with anti-PD-L1 antibody to test our hypothesis that lowering the threshold for antigen signaling can enhance efficacy of the interruption of the PD-1/PD-L1 axis, other researchers may be interested in evaluating combination potential with other approved or emerging immunotherapy agents (e.g., anti-CTLA4, anti-LAG3) in future research.

Previous research has pointed to the involvement of STK17B in immunological processes, as its expression is highly enriched in B and T cells (3). Studies have also shown that STK17B is highly expressed in certain malignancies and is involved in tumor proliferation and metastasis (16–19). STK17B has been suggested as a potential prognostic marker in chronic lymphocytic leukemia, multiple myeloma, and skin cutaneous melanoma (20–22). However, other studies have reported conflicting results, suggesting STK17B’s regulation of apoptosis is dependent on the cellular context (16, 23, 24). The focus of this study was to understand the function of STK17B in T cells, as studies on T cells from STK17B knockout mice have demonstrated a lower threshold for TCR stimulation and that they can respond to suboptimal antigens, pointing to potential utility in cancer immunotherapy (3).

When evaluating a target identified based on genetic knockout data, it is important to assess the potential scaffolding function of the target versus its catalytic activity, which cannot be assessed in knockout animal as the protein is completely absent. Therefore, our first step was to develop potent and selective small molecule inhibitors of STK17B kinase activity. Our findings indicated that the kinase activity of STK17B is fully responsible for its function in T cells, specifically in setting the threshold for TCR activation. We were able to recapitulate the data generated in STK17B-knockout mouse T cells with a small molecule inhibitor *in vitro* and *in vivo.* In addition, we sought to confirm that only STK17B, not its paralog STK17A, is involved in TCR activation pathway of human T cells, as relevant literature is mainly based on data generated in STK17B-knockout mice. Due to a chromosomal rearrangement event, rodents only express *STK17B* (8), whereas humans express both paralogs *STK17A* and *STK17B*. Using STK17A-selective and STK17B-selective compounds, we were able to show that only STK17B inhibition affects human T cells, enhancing IL-2 secretion after TCR activation, while STK17A-selective compounds had no effect on IL-2.

We successfully reproduced the phenotypic changes of T cell function that had been described for STK17B-knockout T cells (3) using our STK17B inhibitor *in vivo* – increased activation markers CD69 as well as increased secretion of proinflammatory cytokines IL-2 and IFN-γ were observed post suboptimal antigen stimulation. In addition, we also confirmed the link between STK17B inhibition and enhanced calcium flux in human T cells *in vitro.* Furthermore, our finding that phosphorylation of myosin light chain (MLC) is mediated by STK17B, and given MLCs role in cell motility, one could speculate that limiting T cell motility by STK17B inhibition can lead to prolonged immunologic synapse formation and thereby explain, together with enhanced calcium flux, how the T cell signaling threshold is lowered such that T cells could be activated with suboptimal antigens.

Next, we demonstrated that pharmacologic inhibition of STK17B translates into enhanced antitumor efficacy in combination with a checkpoint inhibitor in a highly immunogenic syngeneic tumor model (MCA205). However, we did not observe any significant antitumor activity in the less immunogenic MC38 model. As a caveat, the rapid growth of syngeneic tumors compared to human tumors may not allow sufficient time for an immune response to fully manifest in less immunogenic settings. Furthermore, the compounds’ ability to enhance T cell stimulation observed in our *in vitro* studies, under conditions of suboptimal TCR activation, may be limited in the context of an immuno-suppressive tumor microenvironment.

Nevertheless, the development of these potent and selective small molecule tools allowed us to assess whether the kinase function of STK17B is responsible for the T cell activation phenotype, or whether the potential scaffolding functions may also contribute. Such questions cannot be addressed utilizing genetic knockout animals. Beyond that, this study also provides a roadmap to develop assays and methods useful in evaluating the function of kinases as potential cancer immunotherapy targets. As an example, we provide validation data from a suite of target-based and immune functional assays that may be applicable to characterization of other immune-kinases and their inhibitors.

One aforementioned limitation of our approach is that as syngeneic tumor models grow relatively fast, there may have been insufficient time for the STK17B inhibitor-mediated boost of T cell response to take effect. In addition, as knockout mice were given relatively strong stimuli (either with peptide or antibody), lowering the activation threshold alone may not be sufficient in the tumor microenvironment, due to either insufficient tumor antigen, or lack of endogenous immune activation. Furthermore, although our tool molecules are relatively selective for STK17B over its paralog STK17A and the broad kinome, there is still potential for off-target activity through inhibition of other kinases. However, given that our experiments reproduced the known phenotypic changes seen in STK17B knockout mouse T cells, it is most likely that STK17B inhibition is responsible for the majority of immune phenotypes described here. This is corroborated by the fact that our STK17B inhibitor did not have any effect in STK17B-deficient moues T cells. Lastly, our STK17B-selective compounds come from one chemical scaffold, which could limit the generalizability of the findings if there are any inherent issues with this chemical series. Additional research also needs to be done to further understand the mechanistic pathway downstream of STK17B, beyond calcium flux and reduction of myosin light chain phosphorylation, as well as potential long-term phenotypic impact of STK17B deficiency on T cell function.

Our results show that T cell activation can be enhanced under suboptimal conditions via STK17B inhibition. Therefore, it would be useful to investigate whether STK17B inhibitors could be effective as adjuvants in vaccine settings to further boost T cell responses. Future research could also utilize slower growing tumor models to allow sufficient time for the immune system to mount an antitumor immune response in the presence of STK17B inhibitors, alone or in combination with immune checkpoint inhibitors.

In conclusion, this study identified several STK17B inhibitors with demonstrated ability to enhance T cell activation. However, *in vivo* assessment of the highly potent and selective STK17B inhibitor BLU7482 found inconsistent antitumor activities across two mouse syngeneic tumor models. While STK17B has been de-prioritized as a cancer immunotherapy target based on the findings reported here, the methodology and assays validated in this research can be applied to future studies.

## Data Availability

The raw data supporting the conclusions of this article will be made available by the authors, without undue reservation.
