# Supplementary material for: Evaluation of STK17B as a cancer immunotherapy target utilizing highly potent and selective small molecule inhibitors
Source: Front Immunol. 2024 Oct 21;15:1411395. doi: 10.3389/fimmu.2024.1411395 (PMC11536310; doi:10.3389/fimmu.2024.1411395)
Supplement: Supplementary file 1 [file DataSheet1.pdf]

Supplementary material

1 Supplemental methods

1.1 Animals

Female OT-1 mice (C57BL/6-Tg(TCRaTCRb)1100Mjb/J) and C57BL/6 mice were six to eight weeks of age when utilized. After arrival, animals were maintained for one week to become accustomed to the new environment and for observation. Mice were maintained under specific pathogen free conditions with daily cycles of 12 hours light/12 hours darkness according to committed guidelines (GV-Solas; Felasa; TierschG). Health monitoring was carried out on a regular basis. The experimental study protocol was reviewed and approved by the local government (ROB-55.2.2532.Vet_03-20-30).

1.1.1 Mass spectrometry

The protein/peptide sample preparation iST-NHS kit compatible with chemical labeling was from PreOmics (Planegg/Martinsried, Germany). TMT 10plex and TMT (tandem mass tag) 11-131C isobaric label reagents were from Thermo Fisher Scientific (Rockford, IL, USA). AssayMAP Fe(III)-NTA 5 µL cartridges were from Agilent Technologies (Cedar Creek, TX, USA). All other chemicals were from Sigma-Aldrich (Steinheim, Germany) unless otherwise stated.

1.1.2 Protein digestion and peptide tandem mass tag labelling

Mouse T cells ($28x10^6$ per sample) were processed following the manufacturer's instructions with minor modifications. Cells were homogenized by adding LYSE-NHS buffer (100 µL), incubated at 95°C for 10 min and DNA sheared for 20 cycles of alternating bursts and pause (15 sec each) using a cooled sonication device (Bioruptor Plus, Diagenode, Seraing Ougrée, Belgium). Protein concentration was determined using the bicinchoninic assay (BCA, Pierce). Identical protein amounts of each sample (75 µg, 0.94 µg/µL) were transferred to individual cartridges, digested by adding DIGEST solution (80 µL) and incubated at 37°C for two hours. Samples were quenched by adding STOP solution, cartridges washed with WASH1 and WASH2, eluted in a tube with ELUTE, and vacuum centrifuged to dryness.

TMT labeling was performed according to Paulo et al [1]. with minor modifications. Dried peptides were reconstituted in 100 mM HEPES (N-2-hydroxyethylpiperazine-N-2-ethane sulfonic acid) pH 8.5 buffer (ca. 1.5 µg/µL final concentration) and individually labeled with 300 µg TMT 11plex reagents (dissolved in anhydrous acetonitrile, 30% v/v end concentration) at RT for 60 min. The reaction mixtures were quenched with hydroxylamine (0.3% v/v end concentration) at RT for 15 min followed by acidification with formic acid (5% v/v end concentration). Samples were combined and vacuum centrifuged to dryness. Peptides were desalted by SPE (50 mg C18 Sep-Pak, Waters) and eluted with 80% acetonitrile/0.1% trifluoroacetic acid (TFA). Two aliquots (55 and 5 µg) were removed for protein-level analysis and TMT labeling efficiency check, respectively, and vacuum centrifuged to dryness.

1.1.3 Phosphopeptide enrichment

Enrichment of phosphopeptides was performed as described by Post et al [2]. Briefly, phosphorylated (TMT labeled) peptides were enriched using Fe(III)-NTA cartridges in an automated fashion using the AssayMAP Bravo platform (Agilent Technologies). Cartridges were primed with 100 µL of 0.1% TFA in acetonitrile and equilibrated with 50 µL of loading buffer (80% acetonitrile/0.1% TFA). The sample was subsequently split into five (153 µg each, 0.96 µg/µL) and loaded onto five separate cartridges. The columns were subsequently washed with 50 µL of loading buffer, and phosphopeptides were eluted with 25 µL of 1% ammonium hydroxide directly into 25 µL of 10% formic acid solution. Samples were combined and vacuum centrifuged to dryness.

1.1.4 Liquid chromatography-mass spectrometry/mass spectrometry

Liquid chromatography-mass spectrometry (LC-MS/MS) was performed using an EASY-nLC 1200 ultrahigh pressure liquid chromatography (UHPLC) connected to an Orbitrap Fusion Lumos Tribrid and equipped with an EASY-spray source (Thermo Fisher Scientific, San Jose, CA). Samples were re-suspended in 5% formic acid/2% acetonitrile, approximately 1.6 µg sample (total protein) and entire (phospho-enriched) fractions were loaded on an Acclaim PepMap C18 trapping column (75 um×20 mm, 5 um particle size) at a controlled maximum back pressure of 500 bar. Peptides were separated on an Acclaim PepMap C18 EASY-spray column (75 um×750 mm, 2 um particle size) heated at 45°C and at a 270 nl/min flow rate.

The total proteome data were generated using the following gradient: 5% buffer A for 5 min, 5–20% buffer B in 120 min, 20–45% buffer B in 90 min, 45–100% buffer B in 5 min, 100% buffer B for 20 min, corresponding to a total acquisition time of 240 min (buffer A: 0.1% formic acid; buffer B: 0.1% formic acid/80% acetonitrile). The spray voltage used was 1.9-2.2 kV. Mass spectra were acquired using an MS2/MS3 method (also called “multinotch MS3” or Synchronous Precursor Selection, SPS) [3]. The instrument was operated in the data-dependent acquisition (DDA) mode, collecting Orbitrap full MS1 scans over a mass range from m/z 300 to 1400 using quadrupole isolation, a resolution of 120k (at m/z 200), an automatic gain control (AGC) target value of $2x10^5$, and a maximum injection time (IT) of 50 milliseconds (ms). Data were on-the-fly recalibrated using ambient air hexacyclodimethylsiloxane at m/z 445.12002. During a cycle time of three seconds (top speed), the most intense precursor ions, with charge states between 2 and 6, an intensity threshold of $5x10^3$, were mono-isotopically selected for collision induced dissociation (CID), using quadrupole isolation of m/z 0.7, AGC target of $1x10^4$, maximum IT of 50 ms, collision energy of 35%, and ion trap readout with turbo scan rate. Only a single charge state per precursor was selected for MS2. Interrogated precursor ions were dynamically excluded for 75 s using a ±10 ppm mass tolerance.

TMT reporter ions were generated using SPS, quadrupole isolation of m/z 2, high-energy collision dissociation (HCD) at a normalized collision energy of 65%, and readout in the Orbitrap with a resolution of 50k, the scan range of m/z 100 to 500, an AGC target of $5x10^4$, and a maximum IT of 105 ms. The mass range for selecting the SPS (MS3) precursors was from m/z 400 to 2000, excluding the MS2 precursor with a tolerance of m/z 40 (low) and 5 (high), and any TMT neutral loss from it. The number of SPS precursors was set to 10.

Alternatively, the phosphoproteome data were generated using a shorter gradient: 5% buffer B for 5 min, 5–20% buffer B in 60 min, 20–45% buffer B in 60 min, 45–100% buffer B in 5 min, 100% buffer B for 10 min, corresponding to a total acquisition time of 140 min.

MS data were acquired using an MS2-only strategy using HCD and Orbitrap readout at a normalized collision energy of 40%, an intensity threshold of $2.5x10^4$, a scan range with a defined first mass at m/z 100, AGC target of $5x10^4$, and a maximum IT of 250 ms. All the other relevant parameters were essentially the same as previously described.

1.1.5 LC-MS/MS data processing

Raw data were processed with MaxQuant 1.5.6.0 and Andromeda as a search engine. Total proteome and phosphoproteome raw files were defined as separate experiments and processed together using default parameters. Spectra were searched against the UniProt canonical mouse protein database (16,855 entries, downloaded on March 1, 2017), including default contaminants. Total proteome and phosphoproteome files were set as reporter ion MS3 and MS2 type, respectively.

Correction factors for the TMT 10plex and TMT11-131C lot numbers were used as specified in their product data sheet. Phospho (STY) was added as a variable modification for the phosphoproteome experiment. The protein Groups and Phospho (STY) Sites text files were then used for further data analysis with Perseus [4].

2 Supplementary figures and tables

2.1 Supplementary figures

Supplementary figure S1. Correlation between NanoBRET target engagement and enzyme inhibition of STK17A and STK17B.

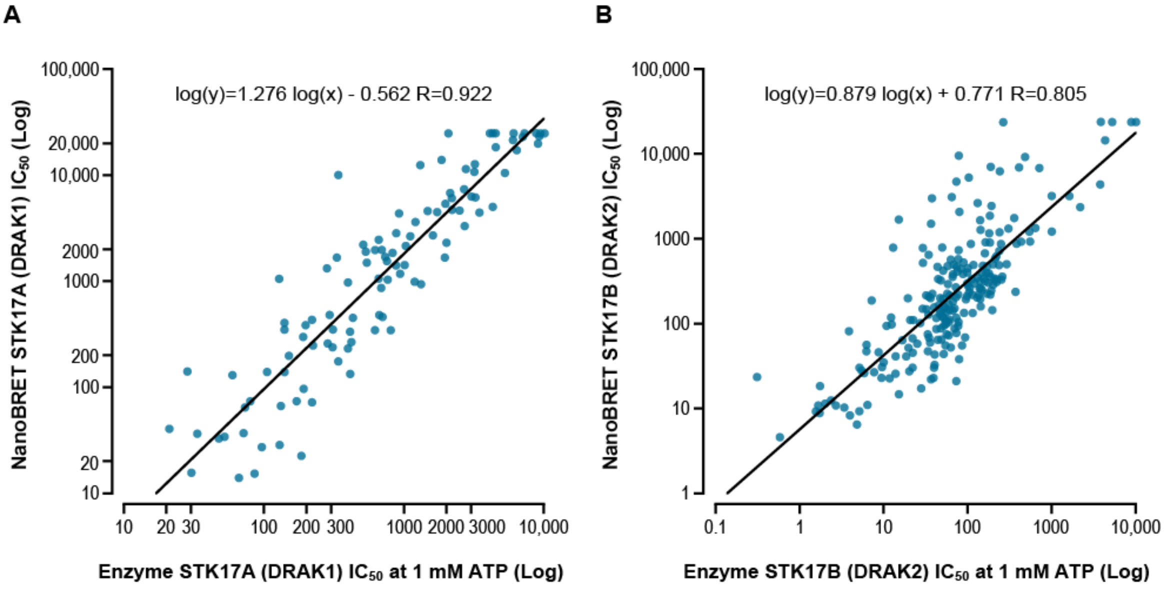

Scatterplots correlating IC_{50} values for STK17A (panel A) and STK17B (panel B) were determined in cellular NanoBRET and enzymatic assays. Custom generated NanoBRET probe, based on one of Blueprint Medicine's proprietary STK17A/B dual inhibitors fused to BODIPY fluorescent dye enabled optimization of NanoBRET target engagement assay. Stable HEK293 cell lines were generated by transfection of FLp-In™ T-REx™ with Nano-luc tag fusion constructs for STK17A and STK17B. Compound target engagement is determined by the displacement of the fluorescent probe measured by BRET.

ATP, adenosine triphosphate; BRET, bioluminescence resonance energy transfer; DRAK1, death-associated protein kinase-related apoptosis-inducing protein kinase 1; DRAK2, death-associated protein kinase-related apoptosis-inducing protein kinase 2; HEK, human embryonic kidney; IC_{50}, half maximal inhibitory concentration.

Supplementary figure S2. Analysis of calcium flux and IL-2 production in STK17B or STK17A inhibitor-treated human T cells.

A

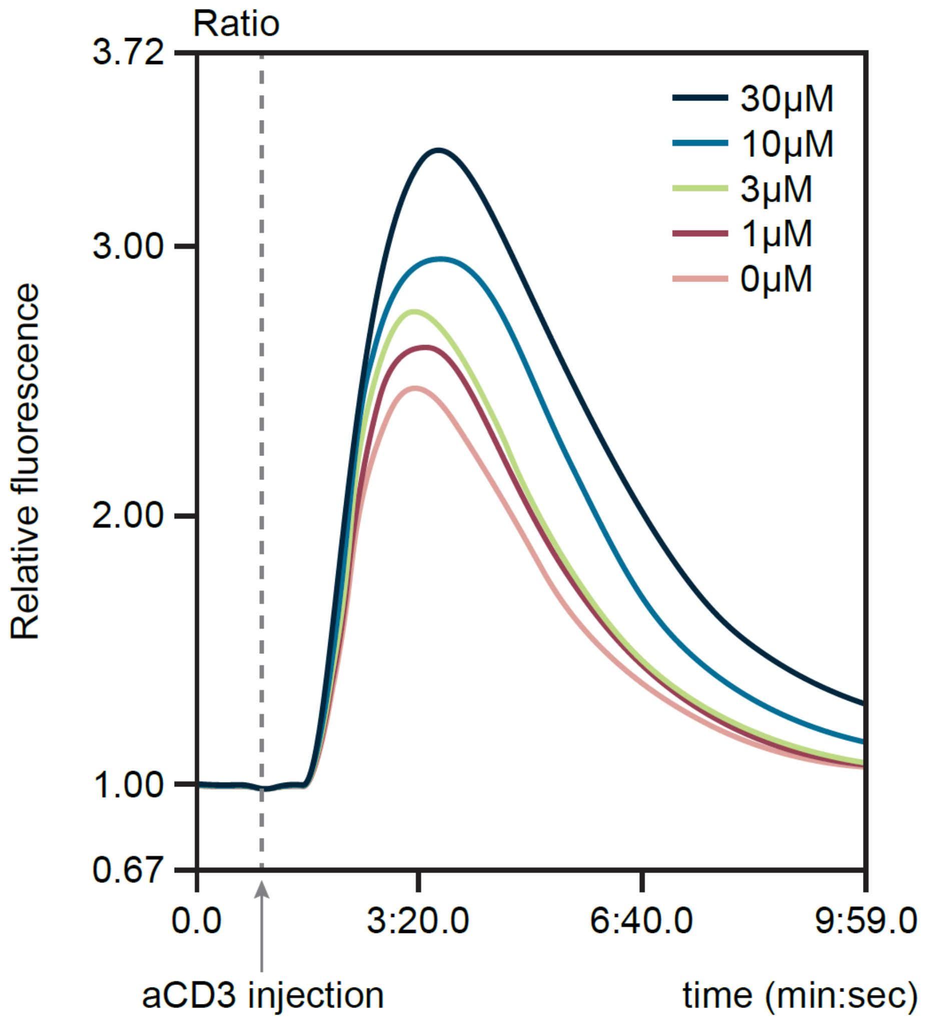

B

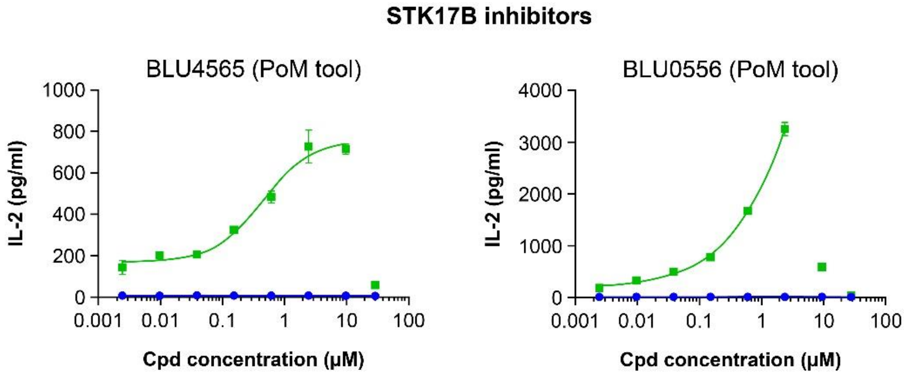

NanoBRET	BLU4565
STK17A (nM)	2300
STK17B (nM)	41

NanoBRET	BLU0556
STK17A (nM)	1130
STK17B (nM)	11

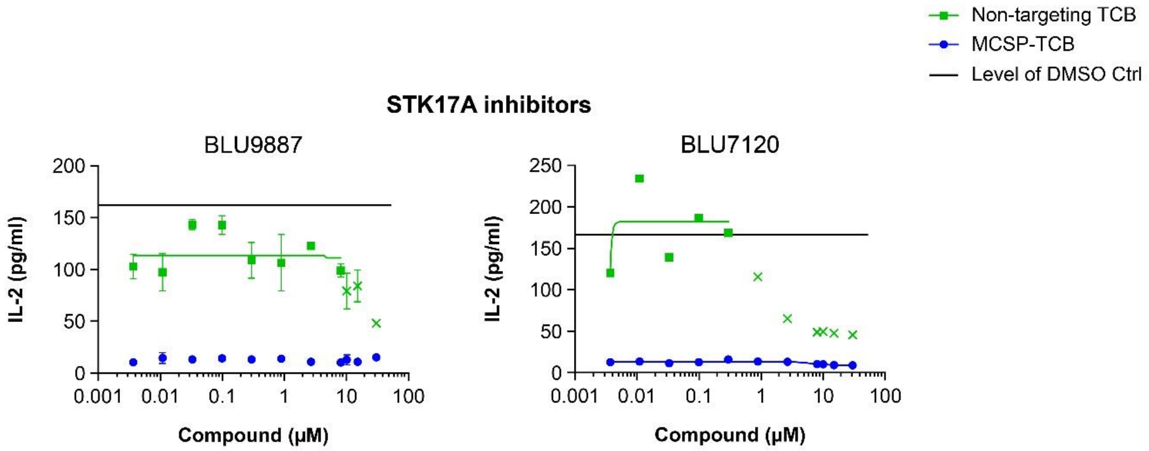

NanoBRET	BLU9887
STK17A (nM)	14
STK17B (nM)	25000

NanoBRET	BLU7120
STK17A (nM)	15
STK17B (nM)	2500

Calcium flux in donor-derived human T cells (A). Isolated human T cells were pretreated with BLU4039 followed by injection of anti-CD3 activating antibody. A dose-dependent increase in the maximal fluorescence units indicated increased Ca2+ flux due to STK17B inhibition.

Primary human T cells were co-cultivated with the melanoma cell line MV3 (B). T cells were treated with increasing concentrations of inhibitors for STK17A and STK17B, respectively. Subsequently, T cells were stimulated using a T cell bispecific antibody crosslinking to MCSP antigen on MV3 cells and CD3 epsilon on T cells. IL-2 in the supernatant was determined 96 hours after T cell stimulation using a cytokine bead array. Data were measured in triplicates.

CD, cluster of differentiation; DMSO, dimethyl sulfoxide; IL-2, interleukin-2; MCSP, melanoma-associated chondroitin sulfate proteoglycan; PoM, proof of mechanism; STK, serine/threonine kinase; TCB, T cell bispecific antibodies.

Supplementary figure S3. Dose-dependent enhancement of proliferation with STK17B inhibitor BLU4039 in WT, but not STK17B KO mouse CD8+ T cells.

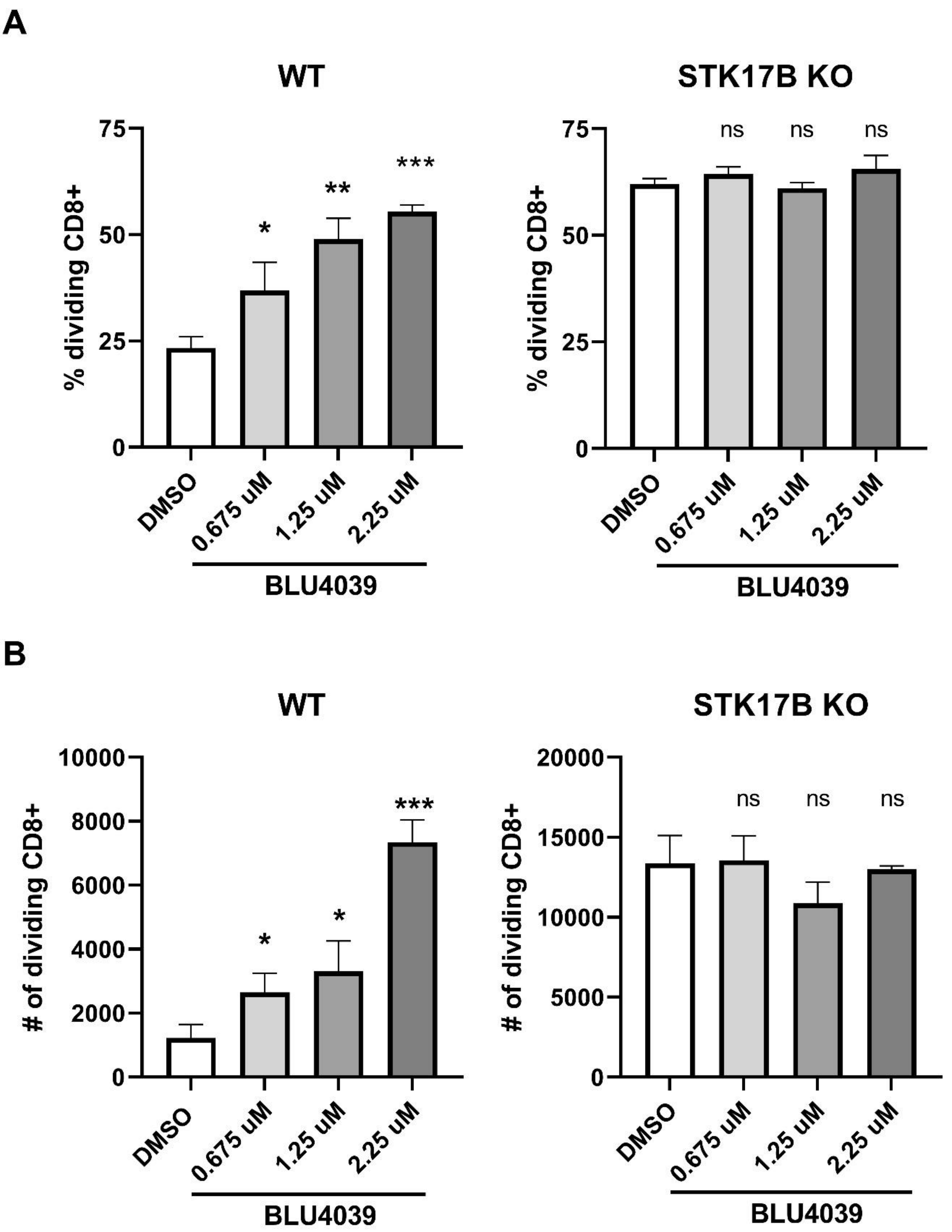

T cells from lymph node and spleen of STK17B knockout mice and WT littermates were purified by negative selection with biotin-conjugated antibodies specific for B220, CD11b, DX5, and MHC class II (eBioscience, San Diego, CA), followed by separation with

streptavidin-conjugated magnetic beads (Miltenyi Biotech, Auburn, CA) on an autoMACS Pro Separator (Miltenyi Biotech). T cells were stained with CFSE (eBioscience) and plated on round-bottom plates that were previously coated with 3 µg/well goat anti-hamster Ig (Vector Laboratories), followed by 250 ng/well anti-CD3 (Clone 2C11, eBioscience) for suboptimal stimulation in the absence or presence of the indicated concentrations of BLU4039. Cells were incubated at 37°C for 72 hours, harvested, and stained with Zombie Red Viability Dye, anti-CD8, and anti-CD4 (BioLegend). The number of live, divided cells was determined by counting each sample for 40 seconds on a Sony SP6800 Spectral Analyzer. % dividing cells (based on CFSE dilution) was determined (A) as well as number of live proliferating cells acquired (B). Unpaired T-test performed in GraphPad PRISM.

CD, cluster of differentiation; CFSE, carboxyfluorescein succinimidyl ester; MHC, major histocompatibility complex; WT, wild type.

Supplementary figure S4. Discovery of STK17B substrate by phosphoproteomics using mouse T cells.

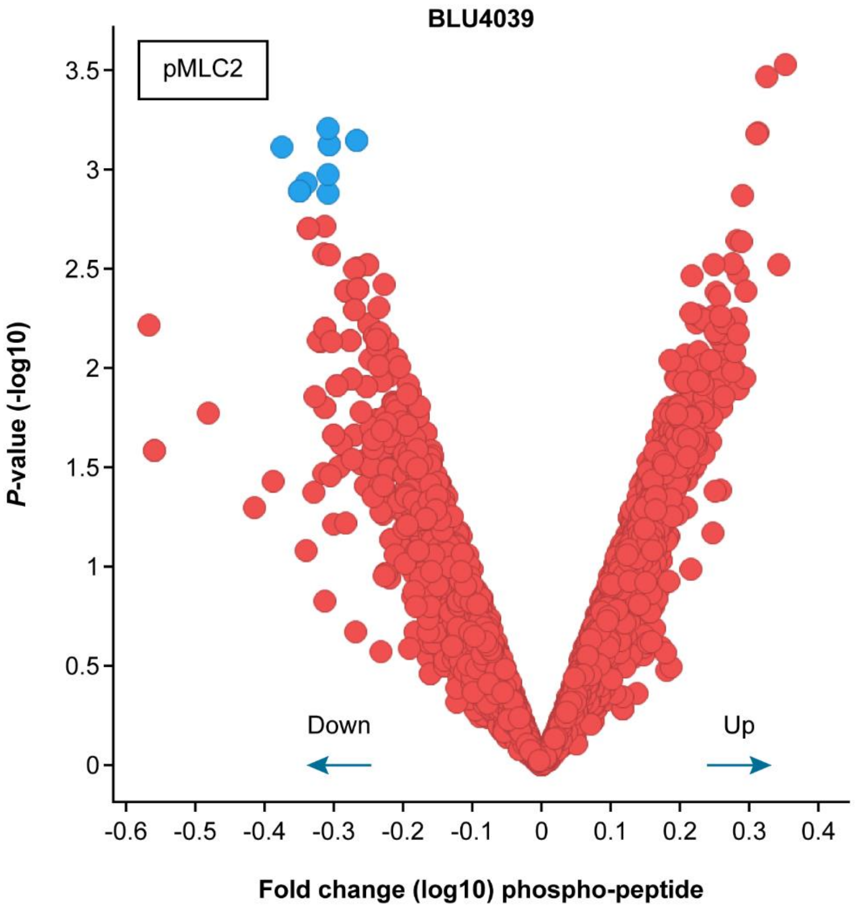

pMLC2 was identified as a substrate for STK17B by quantitative phosphoproteomics. Murine T cells were isolated from splenocytes. T cells were treated in quadruplicates with the STK17B inhibitor BLU4039 at a concentration of 3 µM or DMSO and stimulated with anti-CD3/anti-CD28 and cross-linking secondary antibodies. T cells were lysed and peptides were labelled with an 11-plex TMT kit. Phospho-peptides were enriched via IMAC and labeled samples were pooled. Quantitative phospho-proteomes were identified by LC-MS/MS. Results were plotted as volcano plot with each dot representing one phospho-peptide identified. BLU4039-induced fold changes in phospho-peptide abundance were logarithmically plotted on the x-axis, while the (-log10) p-value was plotted on the y-axis. Peptides down-modulated by compound treatment with a q-value of 5 or lower are plotted in blue.

CD, cluster of differentiation; DMSO, dimethyl sulfoxide; IMAC, immobilized metal affinity chromatography, LC-MS/MS, liquid chromatography-mass spectrometry; pMLC2, phospho-myosin light chain 2; STK, serine/threonine kinase; TMT, tandem mass tag.

Supplementary figure S5. Activity of STK17B inhibitors measured by pMLC2 Flow cytometry assay (A), dose response of tool compounds (B), and correlation with NanoBRET target engagement assay (C).

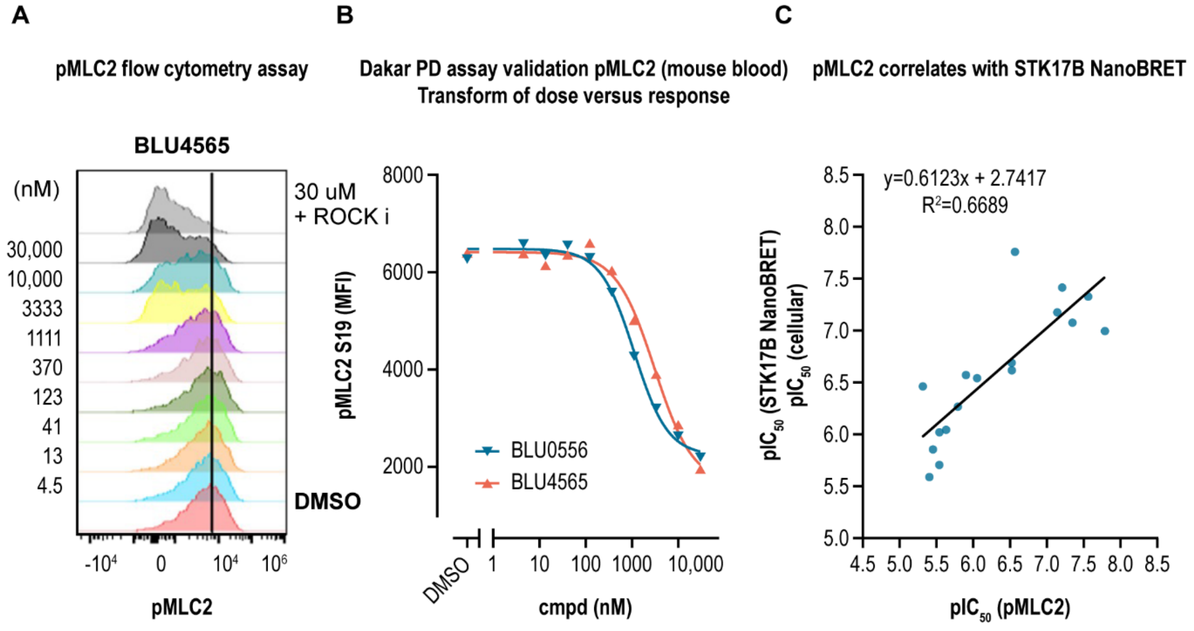

	BLU0556	BLU4565
IC_{50}	1120	3073

Mouse whole blood samples were incubated with STK17B inhibitor BLU4565 for two hours. (A) Subsequently, cells were washed, red blood cells lysed and lymphocytes stained for CD3, fixed and permeabilized for intracellular pMLC2 staining, and analyzed by flow cytometry. ROCK 1/2 inhibitor GSK429286A was added to the top concentration (30 µM) of BLU4565 to determine any additional contribution of these MLC kinases. Minimal further reduction of pMLC2 fluorescence signal was observed. (B) Mouse whole blood samples were treated with indicated concentrations of STK17B inhibitors. Red blood cells were lysed, and lymphocytes stained for CD3, and subsequently fixed and permeabilized for intracellular pMLC2 staining. Dose response curves allowed the determination of IC_{50} values for tool compounds. (C) pMLC2 IC_{50} values correlate with cellular target engagement IC_{50} values.

CD, cluster of differentiation; DMSO, dimethyl sulfoxide; IC_{50}, half maximal inhibitory concentration; PD, pharmacodynamic; pMLC2, phospho-myosin light chain 2; STK, serine/threonine kinase.

Supplementary figure S6. Increased T cell proliferation by STK17B inhibitor treatment *in vivo*.

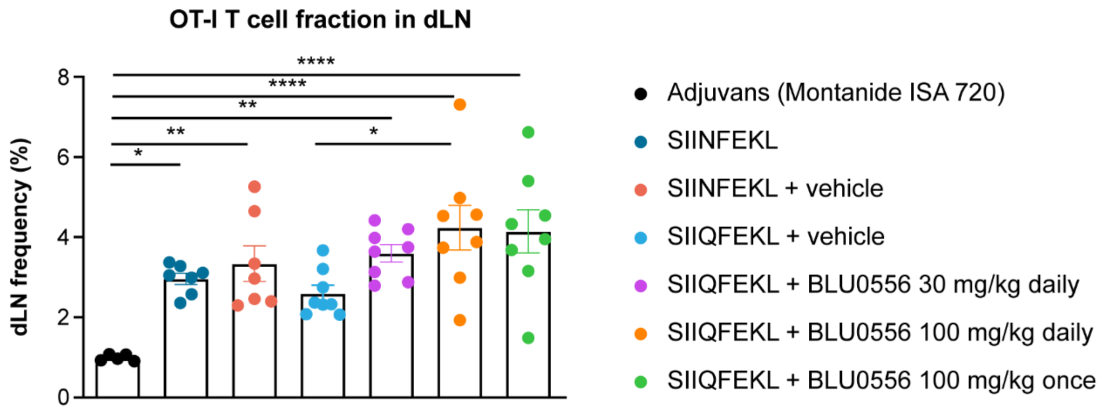

Treatment with STK17B inhibitor increases T cell proliferation after suboptimal antigen receptor stimulation *in vivo*. T cells were isolated from CD45.2+ OT-I TCR transgenic splenocytes. OT-I T cells were transplanted in CD45.1+ mice via tail vein injections (3 million cells per recipient mouse). One day after adoptive OT-I T cell transfer, mice were treated with different doses of STK17B inhibitor (BLU0556). One hour post compound treatment, mice were immunized with 10 µg of an optimal OT-I peptide (SIINFEKL) or a weaker binding mutant (SIIQFEKL). The weaker binding mutant was injected into the mice which received compound treatment. Compound administration continued for two more days until mice were sacrificed. Expansion of OT-I T cells in the draining lymph nodes was determined by flow cytometry staining for CD45.2 positive cells. One-way ANOVA; multiple comparison; Tukey test; *$p < 0.05$, **$p < 0.01$, ***$p < 0.001$, ****$p < 0.0001$.

CD, cluster of differentiation; dLN, draining lymph nodes. STK, serine/threonine kinase; TCR, T cell receptor.

Supplementary figure S7. Effect of BLU7482 on tumor growth in MC-38 mouse model.

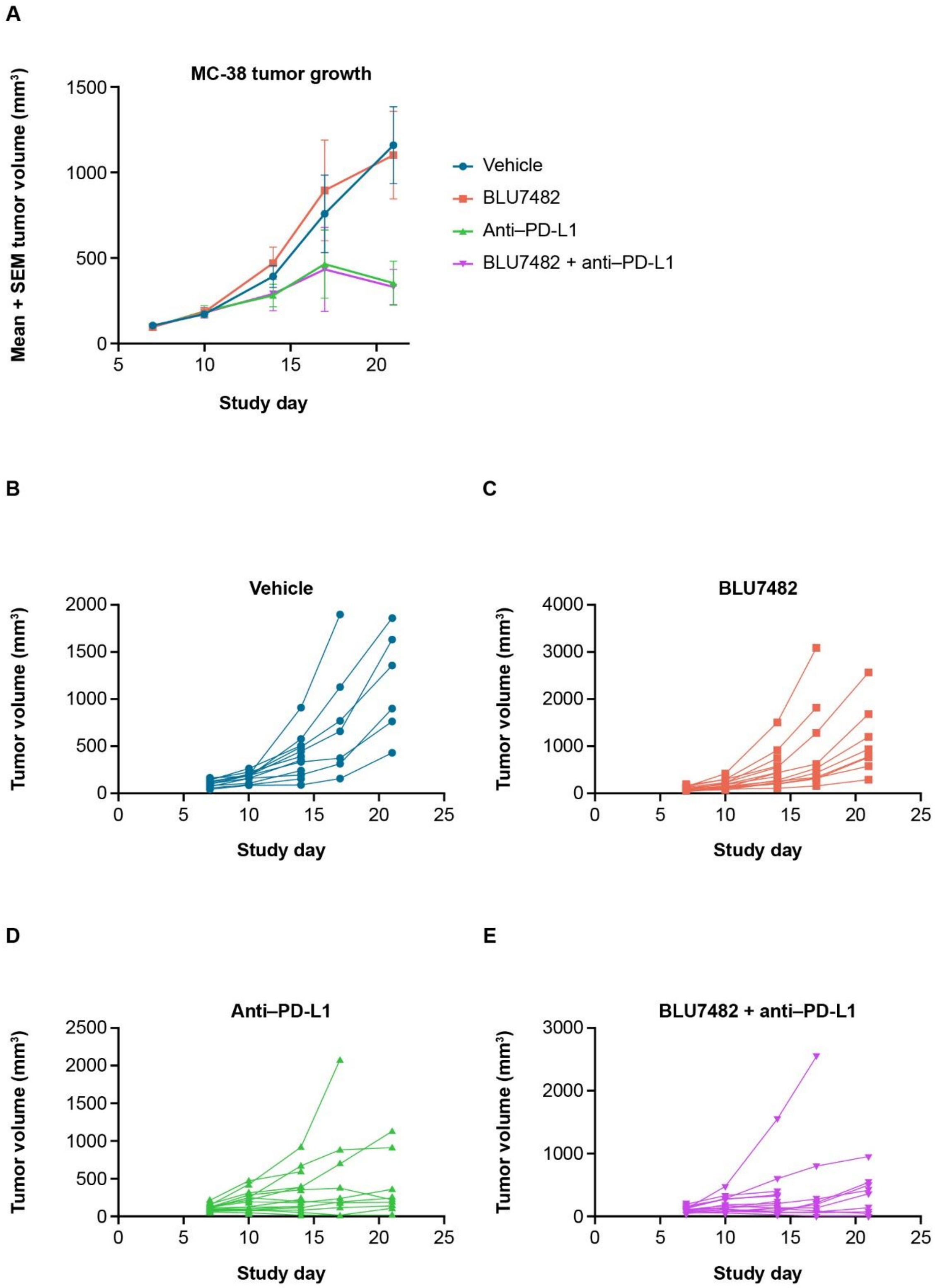

(A) Tumor growth curves of the C57/BL6 MC38 tumor-bearing mice (n = 8 per treatment group) after treatment with BLU7482 (200 mg/kg QD) or with anti–PD-L1 monoclonal antibody alone (10 mg/kg i.p. on the first day of treatment, followed by 5 mg/kg every 3 days until study end), or anti–PD-L1 monoclonal antibody in combination with BLU7482. Vehicle had no treatment. Tumor growth curves for individual MC38 tumor-bearing mice in each treatment group are shown: (B) vehicle, (C) BLU7482, (D) anti–PD-L1, and (E) anti–PD-L1 with BLU7482.

Anti–PD-L1, anti-programmed death-ligand 1 antibody; QD, every day.

Supplementary figure S8. Effect of BLU7482 on tumor growth in MCA205 model with dosing start at tumor implantation (prophylactic dosing)

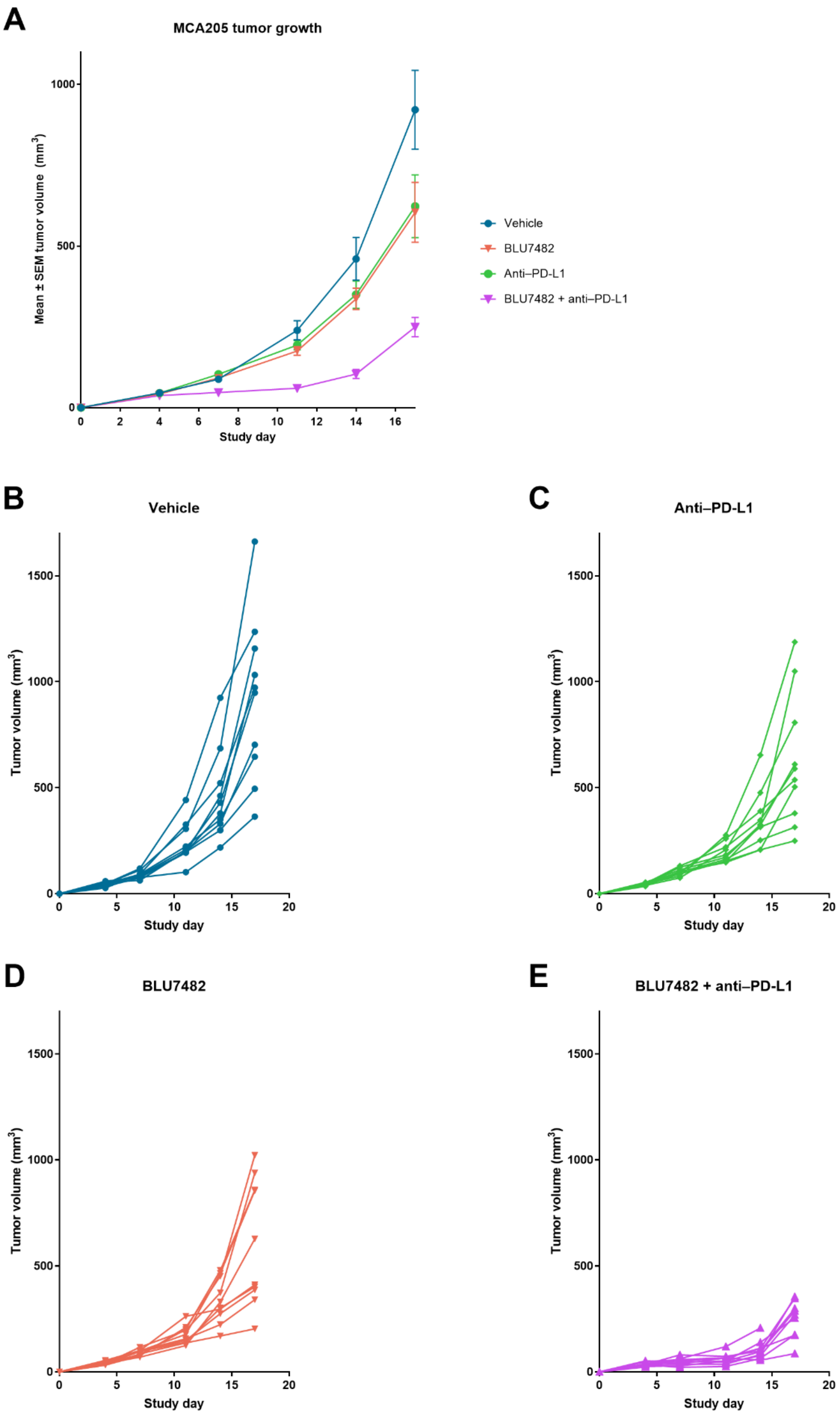

(A) Tumor growth curves of the C57/BL6 MCA205 tumor-bearing mice (n=10 per group) with prophylactic treatment with either BLU7482 (200 mg/kg QD) or anti–PD-L1 monoclonal antibody alone (10 mg/kg i.p. Q3D), or anti–PD-L1 monoclonal antibody in combination with BLU7482. Tumor growth curves for individual MCA205 tumor-bearing mice in each treatment group are shown: (B) vehicle, (C) anti–PD-L1, (D) BLU7482, and (E) anti–PD-L1 with BLU7482.

Supplementary Tables

Supplementary table S1. Enzyme inhibition and NanoBRET target engagement IC_{50} values for selected compounds against STK17A and STK17B.

Compound	S (10) at 3 uM	STK17A enzyme IC_{50} (nM)	STK17B enzyme IC_{50} (nM)	STK17A NanoBRET IC_{50} (nM)	STK17B NanoBRET IC_{50} (nM)
BLU9887	0.089	66.6	5236.9	13.8	>25000
BLU7120	0.055	30.2	163.4	15.4	233.3
BLU4039	0.067	876.9	12.5	2839	101.1
BLU0556	0.107	127.9	2.7	1065	11.1
BLU4565	0.077	2000	22.4	2302	48.6
BLU7482	0.045	45.9	1.0	405.8	2.6

The enzyme inhibition IC_{50} values for selected compounds against STK17A and STK17B measured at 1 mM ATP are shown. NanoBRET target engagement IC_{50} values are also shown.

ATP, adenosine triphosphate; IC_{50}, half maximal inhibitory concentration; NanoBRET, nano-luciferase bioluminescence resonance energy transfer; STK, serine/threonine kinase.

Supplementary table S2. BLU4565, BLU0556, and BLU7482 modulation of IL-2 release.

**Compound**	**IL-2 EC_{50} (nM)**	**OT-1 IC_{50} (nM)**	**IL-2/OT-1 window**
BLU4565	190	3629	19X
BLU0556	35	986	28X
BLU7482	44	6934	163X

EC_{50}, half maximal effective concentration; IC_{50}, half maximal inhibitory concentration; IL-2: interleukin-2.

3 Supplementary references

1. Paulo JA, McAllister FE, Everley RA, Beausoleil SA, Banks AS, Gygi SP. Effects of MEK inhibitors GSK1120212 and PD0325901 in vivo using 10-plex quantitative proteomics and phosphoproteomics. Proteomics 2015;15:462-473.
2. Post H, Penning R, Fitzpatrick MA, Garrigues LB, Wu W, MacGillavry HD, et al. Robust, sensitive, and automated phosphopeptide enrichment optimized for low sample amounts applied to primary hippocampal neurons. J Proteome Res 2017;16:728-737.
3. McAlister GC, Nusinow DP, Jedrychowski MP, Wühr M, Huttlin EL, Erickson BK, et al. MultiNotch MS3 enables accurate, sensitive, and multiplexed detection of differential expression across cancer cell line proteomes. Anal Chem 2014;86:7150-7158.
4. Tyanova S, Temu T, Sinitcyn P, Carlson A, Hein MY, Geiger T, et al. The Perseus computational platform for comprehensive analysis of (prote)omics data. Nat Methods 2016;13:731-740.
